# Networks extracted from nonlinear fMRI connectivity exhibit unique spatial variation and enhanced sensitivity to differences between individuals with schizophrenia and controls

**DOI:** 10.1038/s44220-024-00341-y

**Published:** 2024-11-21

**Authors:** Spencer Kinsey, Katarzyna Kazimierczak, Pablo Andrés Camazón, Jiayu Chen, Tülay Adali, Peter Kochunov, Bhim M. Adhikari, Judith Ford, Theo G. M. van Erp, Mukesh Dhamala, Vince D. Calhoun, Armin Iraji

**Affiliations:** 1 Tri-Institutional Center for Translational Research in Neuroimaging and Data Science (TReNDS), Atlanta, GA USA; 2 Neuroscience Institute, Georgia State University, Atlanta, GA USA; 3 Department of Biological and Medical Psychology, University of Bergen, Bergen, Norway; 4 Department of Child and Adolescent Psychiatry, Institute of Psychiatry and Mental Health, Hospital General Universitario Gregorio Marañón, liSGM, CIBERSAM, School of Medicine, Universidad Complutense, Madrid, Spain; 5 Department of Computer Science and Electrical Engineering, University of Maryland, Baltimore, MD USA; 6 Department of Psychiatry and Behavioral Science, University of Texas Health Science Center at Houston, Houston, TX USA; 7 Department of Psychiatry and Behavioral Sciences, University of California, San Francisco, CA USA; 8 San Francisco Veterans Affairs Medical Center, San Francisco, CA USA; 9 Clinical Translational Neuroscience Laboratory, Department of Psychiatry and Human Behavior, University of California, Irvine, CA USA; 10 Department of Physics and Astronomy, Georgia State University, Atlanta, GA USA; 11 Department of Computer Science, Georgia State University, Atlanta, GA USA

**Keywords:** Schizophrenia, Network models

## Abstract

Schizophrenia is a chronic brain disorder associated with widespread alterations in functional brain connectivity. Although data-driven approaches such as independent component analysis are often used to study how schizophrenia impacts linearly connected networks, alterations within the underlying nonlinear functional connectivity structure remain largely unknown. Here we report the analysis of networks from explicitly nonlinear functional magnetic resonance imaging connectivity in a case–control dataset. We found systematic spatial variation, with higher nonlinear weight within core regions, suggesting that linear analyses underestimate functional connectivity within network centers. We also found that a unique nonlinear network incorporating default-mode, cingulo-opercular and central executive regions exhibits hypoconnectivity in schizophrenia, indicating that typically hidden connectivity patterns may reflect inefficient network integration in psychosis. Moreover, nonlinear networks including those previously implicated in auditory, linguistic and self-referential cognition exhibit heightened statistical sensitivity to schizophrenia diagnosis, collectively underscoring the potential of our methodology to resolve complex brain phenomena and transform clinical connectivity analysis.

## Abstract (web summary)

Analysis of neuroimaging data of people with schizophrenia and healthy controls shows that networks derived from explicitly nonlinear whole-brain functional connectivity exhibit higher reliability than those extracted from linear whole-brain functional connectivity and demonstrate higher sensitivity to the diagnosis of schizophrenia.

## Main

Schizophrenia is a brain disorder thought to be underpinned by altered neural interactions at various spatial and temporal scales^1^. At the whole-brain level, functional magnetic resonance imaging (fMRI) functional connectivity (FC) analysis is a non-invasive approach that has commonly been used to study how schizophrenia-related brain alterations are reflected within statistical relationships between blood-oxygenation-level-dependent (BOLD) time series. Although the relationship between the BOLD signal and neural activity is indirect^2^, experimentally induced and resting-state BOLD fluctuations are typically associated with changes in local field potentials across multiple frequency bands^3–6^, indicating that fMRI FC analysis is a promising tool for advancing the identification of task-related and spontaneously emerging networks of interacting brain regions. Moreover, fMRI FC analysis is deployable within a wide range of predictive clinical contexts. For example, multiple large-scale meta-analyses have shown that FC measures reliably distinguish healthy controls (HC) from individuals with schizophrenia (SZ)^7–9^. Such studies have contributed to an accumulation of evidence for the SZ ‘dysconnection hypothesis’^10^, which posits FC alteration as a central endophenotype of the disorder resulting from neuromodulatory and synaptic pathogenesis.

However, FC studies are typically designed to estimate networks that reflect linear statistical relationships between brain areas^11–13^. Although the remarkably complex nonlinear interactions inherent to brain networks have been recognized and investigated^14–18^, there is a need to develop data-driven methods capable of estimating networks that accurately reflect the structure of these nonlinear connectivity patterns^19^ and thus fill the gap in knowledge concerning their contributions to brain function and alterations in psychiatric disorders such as SZ. In this Article we highlight three ways in which decomposing nonlinear brain connectivity patterns into data-driven networks has the potential to advance systems, cognitive and predictive clinical neuroscientific research. First, effectively capturing networks from nonlinear patterns in a data-driven fashion may lead to a more precise and thorough characterization of the organization and dynamics of neural ensembles at multiple scales^16,20,21^. Second, networks that accurately reflect underlying nonlinear connectivity patterns may reveal unique associations with cognitive and behavioral capacities. In principle, nonlinearity is thought to underpin a high-dimensional state space capable of supporting a set of flexible and diverse neural computations^15,17^, such that analyzing the functional role of nonlinear encoding of information^22^ may shed light on the structure of cognitive processes and deficiencies associated with psychiatric disorders such as SZ and their symptoms. Third, networks captured from measures that are sensitive to nonlinearity can be leveraged to develop biomarkers that can be incorporated within brain-based predictive models of mental illness, or ‘predictomes’^23^.

Among the available network estimation methods, independent component analysis (ICA) is known to be a powerful multivariate source separation technique^24,25^. ICA assumes that the data are a linear mixture of statistically independent source signals and aims to estimate an unmixing matrix, yielding components that approximate these signals optimally^24,25^. In the context of fMRI FC analysis, spatial ICA has commonly been used to decompose fMRI time-series data into a set of intrinsic connectivity networks (ICNs), where the spatial pattern of a network describes its distribution across voxels and the temporal pattern describes its activity over time^11,26–28^. ICNs can be robustly and consistently identified from both resting-state^26,27,29^ fMRI (rsfMRI) and task-based^11,26,30,31^ fMRI (tfMRI) time-series data at different spatial scales^20,21,32^. ICNs can also be reliably extracted from FC matrices constructed from second-order statistics such as Pearson correlation (that is, from the connectivity domain)^33,34^. Connectivity-domain ICA is a type of feature-based analysis^35^ that yields cross-validating components, and it is distinguished from time-domain ICA by unique benefits such as consistency across changes in particular analysis parameters and reproducibility^33^. Moreover, an expanding range of FC metrics can be used to construct the connectivity basis, making connectivity-domain ICA an incredibly versatile tool^33^.

**Fig. 1:**
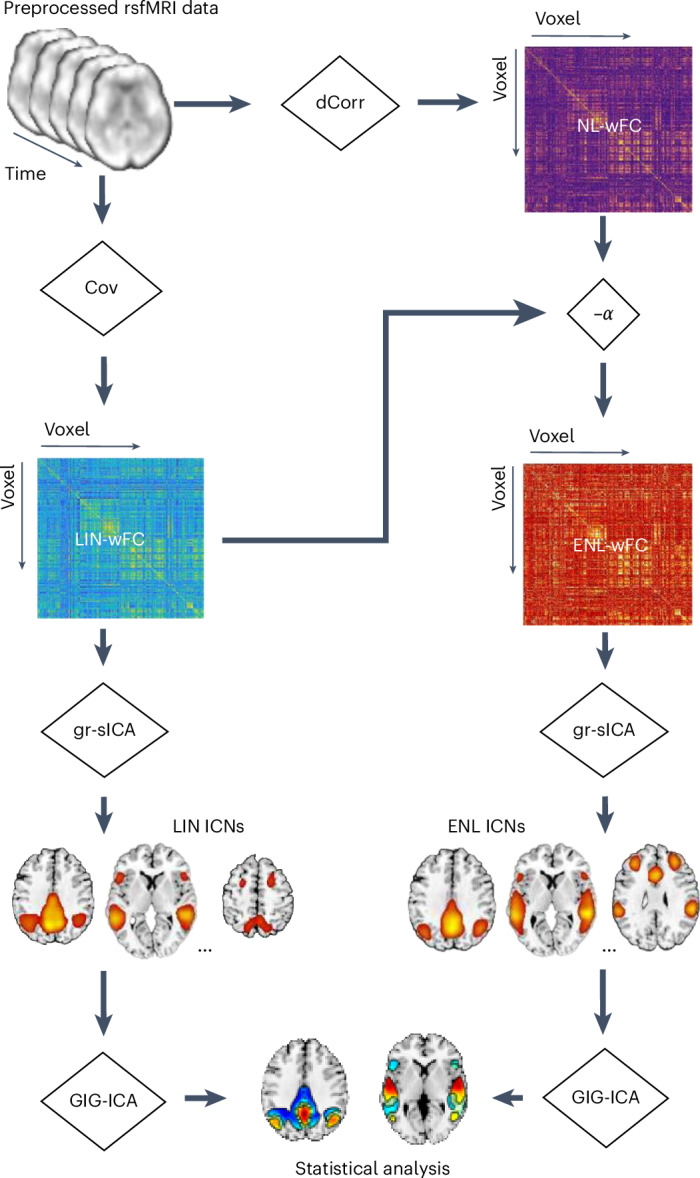
Schematic of the analysis pipeline. Preprocessed rsfMRI data are transformed to the connectivity domain using Cov (as a linear FC estimator) and dCorr as a nonlinear FC estimator. ENL-wFC is obtained by removing the NL-wFC information which is linearly explained by LIN-wFC. Gr-sICA is implemented in the connectivity domain on LIN-wFC and ENL-wFC to estimate separate sets of intrinsic connectivity networks (LIN and ENL ICNs). GIG-ICA is then used to estimate subject-specific ICNs, and statistical analysis is conducted on the subject-level spatial maps.

**Fig. 2:**
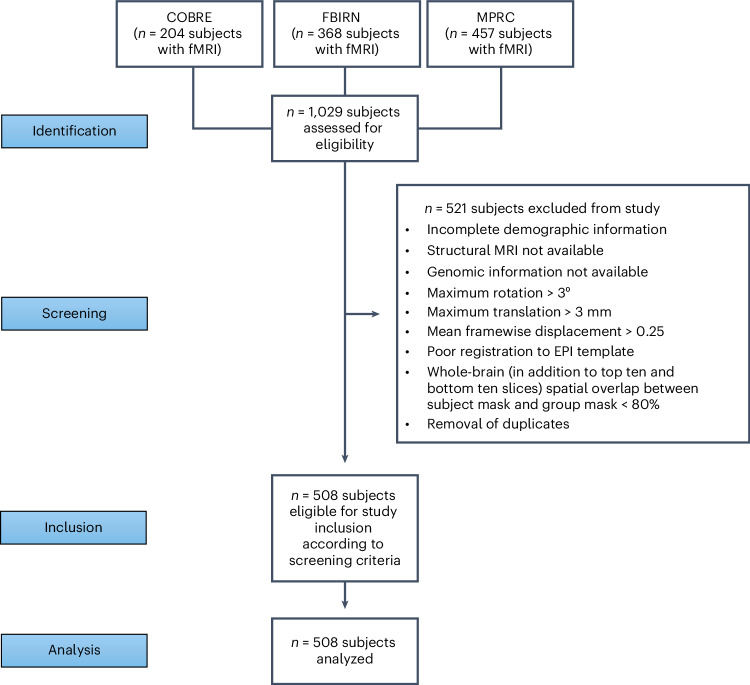
Strengthening the reporting of observational studies in epidemiology (STROBE) flowchart. COBRE, Center for Biomedical Research Excellence; FBIRN, Functional Imaging Biomedical Informatics Research Network; MPRC, Maryland Psychiatric Research Center. EPI, echo-planar imaging.

Connectivity- and time-domain ICA have become valuable tools for investigating fMRI data. However, both methods are typically designed to identify ICNs composed of covarying brain regions, thereby capturing ensembles explained by linear connectivity information^11,33,34^. Although recent advancements have made strides in incorporating nonlinearity, such as learning local spatial or temporal nonlinear structures^36,37^, the extent to which the estimated sources reflect nonlinear connectivity patterns remains unclear. To address this gap in knowledge, we advance an approach to extract ICNs from distance correlation^38^ patterns that move beyond those constructed from Pearson correlation (Fig. 1). We first estimate explicitly nonlinear whole-brain FC (ENL-wFC) by using a linear regression-based approach to remove the nonlinear whole-brain FC information (NL-wFC; operationalized as distance correlation) explained by linear whole-brain FC (LIN-wFC), and we subsequently implement group-level spatial ICA (gr-sICA) in the connectivity domain^39^, resulting in a targeted analysis of network features that are inaccessible to approaches that aim to compute brain connectivity using methods that incorporate both linear and nonlinear information. Although alternate metrics can be used to quantify fMRI connectivity while accounting for higher-order statistics^40–42^, distance correlation is a powerful and flexible dependence metric that remains underexplored in the context of FC research. Moreover, the proposed method is unique, in that we conceive of ENL-wFC as a global feature of the connectivity space rather than as a composite feature constructed from pairwise associations^41,42^. This allows us to leverage information present within global connectivity features beyond those found within macroscopic linear connectivity patterns. In this Article we use this approach to assess differences in spatial variation between explicitly nonlinear (ENL) and linear (LIN) network estimates and to investigate SZ-associated network alterations in a multi-study rsfMRI dataset sourced from three major psychosis projects: the Center for Biomedical Research Excellence (COBRE)^43^, the Functional Imaging Biomedical Informatics Research Network (FBIRN)^44,45^ and the Maryland Psychiatric Research Center (MPRC) (Fig. 2 and Table 1)^46^.

**Table 1:** Subject demographic information

Dataset	Diagnosis (no.)	Sex (no.)	Race (AMR/EUR/AFR/other)	Age (years)^a^	Age (years)^b^
COBRE	HC (75)	Male (56)	20/32/4/0	39.27 ± 12.13	39/(18–65)
		Female (19)	13/3/3/0	35.47 ± 10.02	34/(18–58)
	SZ (51)	Male (45)	19/23/3/0	37.36 ± 15.28	33/(19–64)
		Female (6)	4/1/1/0	40.83 ± 17.70	44/(20–65)
FBIRN	HC (88)	Male (60)	12/48/0/0	36.58 ± 10.74	39/(19–59)
		Female (28)	6/22/0/0	36.61 ± 11.07	33/(19–58)
	SZ (60)	Male (52)	17/35/0/0	39.54 ± 11.12	41/(18–60)
		Female (8)	1/7/0/0	35.88 ± 9.85	34/(24–51)
MPRC	HC (152)	Male (69)	6/43/19/1	38.99 ± 13.22	41/(18–68)
		Female (83)	8/44/30/1	39.93 ± 14.93	43/(16–64)
	SZ (82)	Male (57)	3/33/19/2	36.25 ± 13.54	33/(13–63)
		Female (25)	0/13/11/1	44.68 ± 11.92	47/(13–61)

COBRE, Center for Biomedical Research Excellence. FBIRN, Functional Imaging Biomedical Informatics Research Network. MPRC, Maryland Psychiatric Research Center. HC, healthy control. SZ, schizophrenia. AMR, mixed American. EUR, European. AFR, African.

^a^Mean ± s.d.

^b^Median/range.

## Results

### Goodness of fit

Goodness-of-fit statistics (*R*^2^) for the linear regression of NL-wFC on LIN-wFC were as follows: mean ± s.d. = 0.5337 ± 0.2009; minimum–maximum = 0.0173–0.9413. This indicates that, on average, much of the NL-wFC variance is captured by a linear fit. After accounting for confounding factors (Methods), *R*^2^ is significantly higher for HC versus SZ (*n* = 508; *P* = 0.0002, observed difference = 0.1203, Hedges’s *g* = 0.6971). The observed Hedges’s *g* value indicates the presence of a medium to large effect size. HC residual indices were mean ± s.d. = 0.0457 ± 0.1660; minimum–maximum = −0.4912–0.4936. SZ residual indices were mean ± s.d. = −0.0746 ± 0.1827; minimum–maximum = −0.4725–0.3800.

### Component estimation reliability is greater for ENL versus LIN

Components estimated from ENL-wFC exhibit significantly higher estimation reliability (ICASSO IQ) compared to components estimated from LIN-wFC (*n* = 40 components; *P* = 0.0006, observed difference = 0.037, Hedges’s *g* = 0.6441). The observed Hedges’s *g*-value indicates the presence of a medium to large effect size. ENL stability indices were mean ± s.d. = 0.9694 ± 0.0057; minimum–maximum = 0.9579–0.9800. LIN stability indices were mean ± s.d. = 0.9324 ± 0.0810; minimum–maximum = 0.6186–0.9770.

### Common and unique ICNs identified from ENL and LIN

**Fig. 3:**
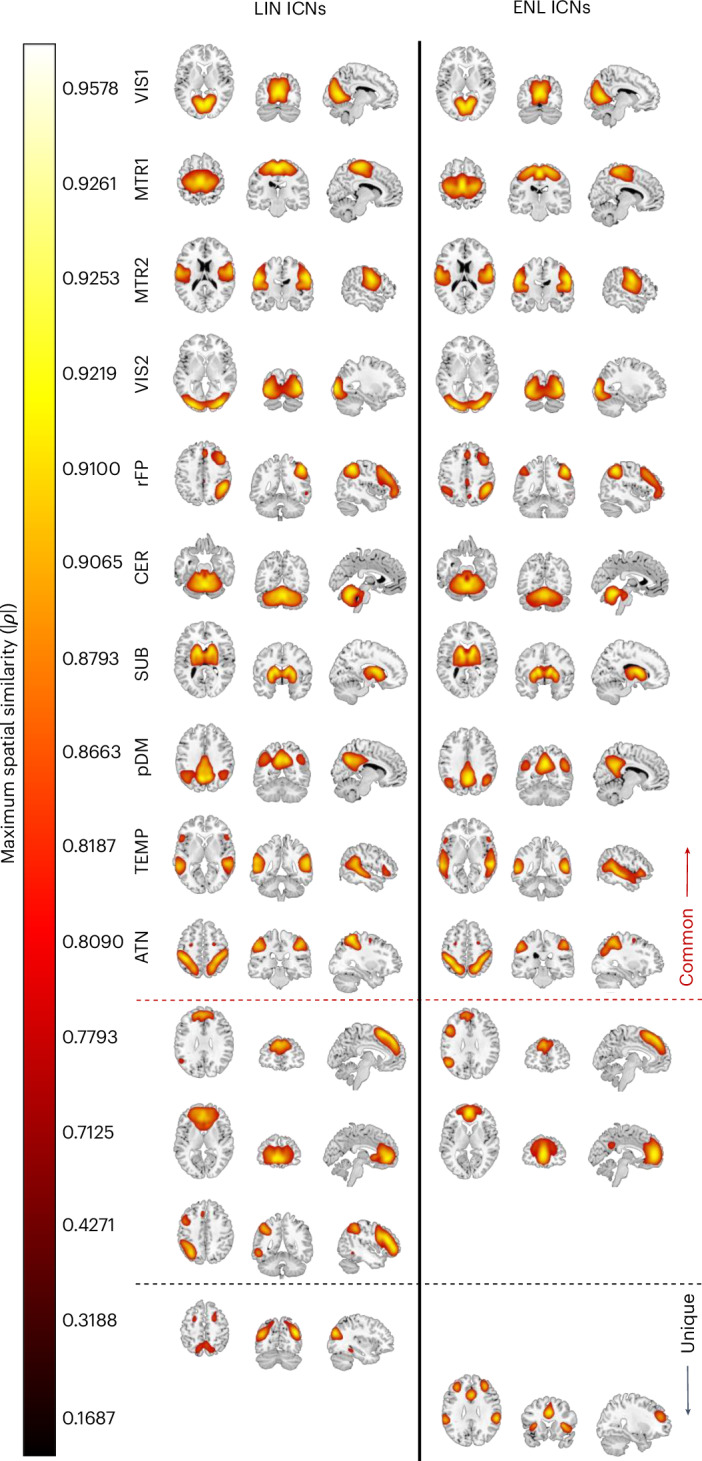
ICNs obtained from LIN-wFC and ENL-wFC gr-sICA in the connectivity domain. ICNs are displayed using an empirical threshold (*Z* > 1.96; *P* < 0.05) on the ch2bet template in order of maximum spatial similarity. Common ICNs (maximum similarity > 0.80) include primary visual (VIS1), primary sensorimotor (MTR1), secondary sensorimotor (MTR2), secondary visual (VIS2), right frontoparietal (rFP), cerebellum (CER), subcortical (SUB), posterior default mode (pDM), temporal (TEMP) and dorsal attention (ATN). ICNs exhibiting maximum similarity between 0.40 and 0.80 and unique ICNs (maximum similarity < 0.40) are also displayed.

Within our 20-model-order gr-sICA framework, 13 ENL ICNs and 14 LIN ICNs were identified (Fig. 3). Among the identified networks, ten exhibited maximum spatial similarity values exceeding 0.80 between their ENL and LIN estimates. We classified these networks as common to both ENL-wFC and LIN-wFC based on the defined criterion (Methods). Among those remaining, two ENL and three LIN ICNs exhibited maximum spatial similarity values between 0.40 and 0.80. Although several of these networks attained relatively high maximum spatial similarity, we noticed distinct intensity differences across their neuroanatomical distributions that prevented common classification and labeling. Furthermore, our analysis uncovered a LIN network and an ENL network exhibiting a maximum spatial similarity less than 0.40. We classified these networks as unique based on our uniqueness criterion (Methods), and we validated the uniqueness of the ENL ICN in question across 100 additional iterations of gr-sICA (Supplementary Note 1 and Supplementary Fig. 1).

### ENL and LIN ICNs exhibit unique spatial patterns

**Fig. 4:**
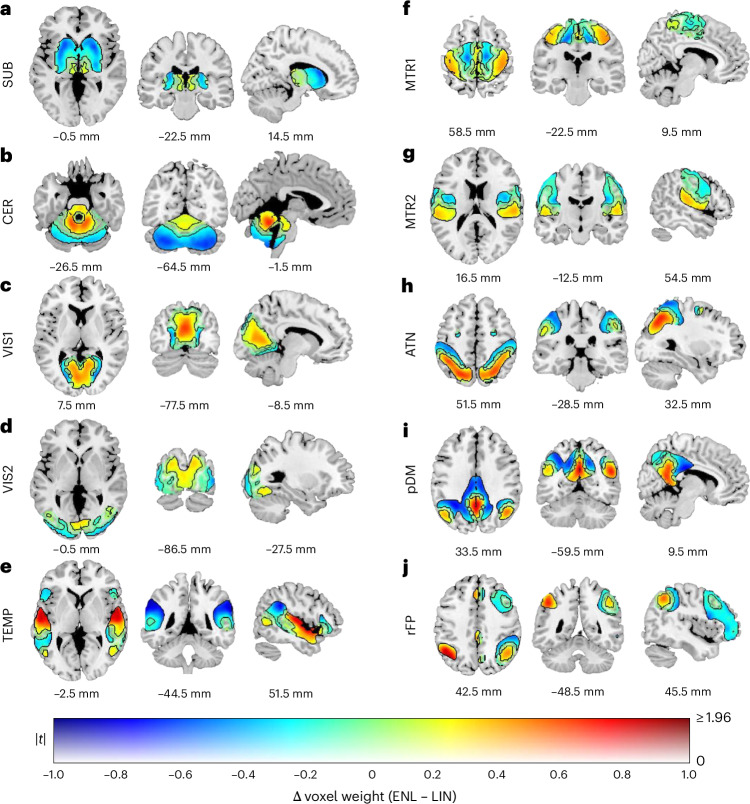
Assessment of ICN spatial variation. Results are plotted according to a dual-coded^86^ colormap, with transparency reflecting two-sided paired sample *t*-statistic magnitudes and contours indicating FDR-corrected statistical significance (*q* < 0.05). Warmer hues indicate ENL > LIN, and cooler hues indicate LIN > ENL. **a**–**j**, The displayed ICNs are subcortical (SUB) (**a**), cerebellum (CER) (**b**), primary (VIS1) (**c**) and secondary (VIS2) (**d**) visual, temporal (TEMP) (**e**), primary (MTR1) (**f**) and secondary (MTR2) (**g**) sensorimotor, dorsal attention (ATN) (**h**), posterior default mode (pDM) (**i**) and right frontoparietal (rFP) (**j**). The results are overlaid on the ch2bet template with *x*, *y* and *z* coordinates listed relative to the origin in Montreal Neurological Institute 152 space.

ENL and LIN ICNs exhibit distinctive spatial distributions (Fig. 4a–j). Visible gradients are present within networks associated with both lower and higher cognitive functioning, and many core regions (defined as regions that attain higher values across the spatial distribution) exhibit greater ENL weight. For the subcortical (SUB) network (Fig. 4a), LIN weight is greater within the bilateral caudate and putamen, and ENL weight is greater within the bilateral thalamus. The cerebellum (CER; Fig. 4b) exhibits higher ENL weight within vermis lobules I–V, and higher LIN weight within lobules VII–IX and the bilateral hemisphere. Among networks associated with visual^47^ and auditory and linguistic^48^ functioning, ENL weight is predominantly greater within spatially central regions, whereas LIN weight is greater within peripheral areas. For instance, the primary visual (VIS1) network (Fig. 4c) exhibits a medial–lateral gradient in the bilateral cortex surrounding the calcarine fissure, with greater ENL weight within the cuneus. The secondary visual (VIS2) network (Fig. 4d) shows higher ENL weight within the cuneus and higher LIN weight within the bilateral inferior and middle occipital gyri. Temporal (TEMP) network (Fig. 4e) variation follows a similar center–periphery pattern, with greater ENL weight in the superior temporal gyri and greater LIN weight within the supramarginal gyri and bilateral inferior frontal triangularis.

Whereas both the primary and secondary sensorimotor networks (MTR1 and MTR2) exhibit gradients (Fig. 4f–g), MTR1 comparisons reveal a medial–lateral pattern between the paracentral lobules and pre- and postcentral gyri, while MTR2 comparisons reveal an inferior–superior gradient between the superior temporal lobe and pre- and postcentral gyri. Networks implicated in higher cognitive functions such as attention^49^, social cognition and self-referential processes^50^, and executive control^51^ exhibit core–periphery gradients. The dorsal attention (ATN; Fig. 4h) network shows higher ENL weight in the superior parietal lobules and higher LIN weight in the postcentral gyri. The posterior default mode (pDM) network (Fig. 4i) exhibits higher ENL values in the precuneus and bilateral angular gyri, with higher LIN values in the middle and posterior cingulate. The right frontoparietal (rFP) network (Fig. 4j) exhibits higher ENL values within the angular gyri (particularly within the left angular gyrus) and higher LIN values within the right inferior parietal lobule, right middle frontal gyrus and right inferior frontal triangularis. The robustness of our voxel-wise *t-*test assessment of spatial variation was confirmed by permutation test results from pDM comparisons (*ρ* = 0.9653). Summary test information for the spatial variation analysis is provided in Supplementary Table 1.

### ENL ICN voxels exhibit enhanced sensitivity to SZ diagnosis

**Fig. 5:**
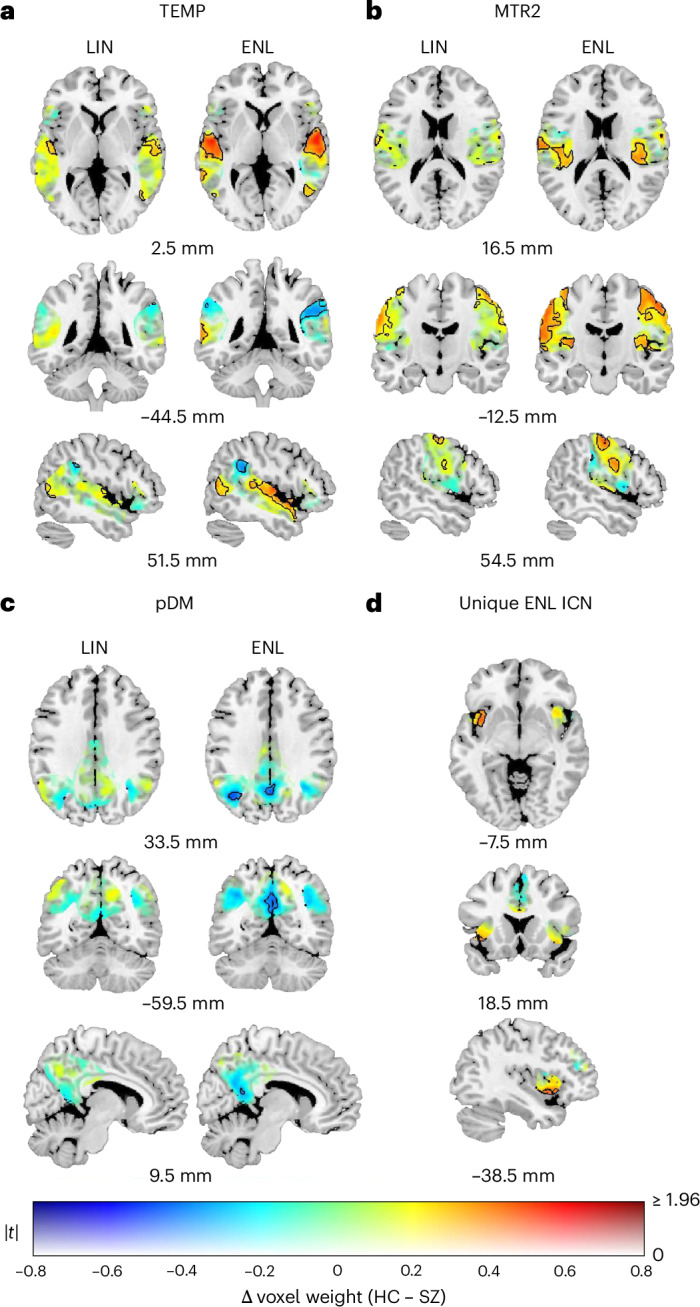
Statistical comparisons between subject-level ICN estimates from healthy controls and individuals with schizophrenia. **a**–**d**, Comparisons between subject-level temporal (TEMP) (**a**), secondary sensorimotor (MTR2) (**b**), posterior default mode (pDM) (**c**) and unique explicitly nonlinear (ENL) (**d**) ICN estimates derived from healthy controls (HC) and individuals with schizophrenia (SZ). Results are plotted according to a dual-coded colormap^86^, with transparency reflecting two-sided independent-samples *t*-statistic magnitudes and contours indicating FDR-corrected statistical significance (*q* < 0.05). In **a**–**c**, results from LIN comparisons are located on the left, and results from ENL comparisons are located on the right. Warmer hues indicate HC > SZ, and cooler hues indicate SZ > HC. Results are overlaid on the ch2bet template with *x*, *y* and *x* coordinates listed relative to the origin in Montreal Neurological Institute 152 space.

Collectively, ENL network voxels exhibit a greater degree of sensitivity to SZ diagnosis versus LIN (*χ*^2^ = 53.75; *P* < 0.00001; odds ratio = 1.24), and a greater number of ENL voxels are implicated (Supplementary Table 2). Moreover, ENL counterparts of networks implicated in auditory and linguistic^48,52,53^, sensorimotor^54^ and self-referential^50^ cognitive processes exhibit enhanced sensitivity to differences between HC and SZ (Fig. 5a–c). For example, although both sets of comparisons revealed differences within TEMP regions comprising the primary auditory and auditory association cortex, ENL comparisons are more sensitive (*χ*^2^ = 851.3; *P* < 0.00001; odds ratio = 22.63), revealing clusters that are more numerous, with augmented volumes and effect sizes (Fig. 5a). LIN and ENL tests revealed higher values for HC within the bilateral superior temporal gyri and temporal poles, bilateral insula, bilateral Heschl’s gyrus, bilateral Rolandic operculum and right middle temporal gyrus, along with higher values for SZ within the right supramarginal gyrus. However, ENL tests revealed a larger number of significant voxels across these regions. Additionally, ENL tests revealed higher HC values within the left middle temporal gyrus and higher SZ values within the left supramarginal gyrus, both of which were missed for significance by LIN tests.

ENL sensitivity was also greater for MTR2 (Fig. 5b; *χ*^2^ = 639.5; *P* < 0.00001; odds ratio = 7.61) and pDM (Fig. 5c; *χ*^2^ = 125.03; *P* < 0.00001; odds ratio = 128) tests. Both sets of MTR2 comparisons revealed greater weight for HC within the bilateral postcentral gyri. However, ENL tests revealed more extensive clusters and greater HC values within the bilateral posterior insula. ENL pDM tests revealed clusters of higher values for SZ within the precuneus and left angular gyrus, whereas LIN comparisons identified only two significant voxels. However, we note that LIN tests were more sensitive for CER (*χ*^2^ = 445.7; *P* < 0.00001; odds ratio = 3.93), VIS1 (*χ*^2^ = 76.85; *P* < 0.00001; odds ratio = 2.28), VIS2 (*χ*^2^ = 391; *P* < 0.00001; odds ratio = 394), MTR1 (*χ*^2^ = 6.868; *P* = 0.0088; odds ratio = 1.76) and rFP (exact binomial test; *P* < 0.00001; odds ratio undefined) networks. Unique LIN network comparisons failed to reveal any group differences (Supplementary Fig. 2h), but unique ENL network comparisons revealed a cluster within the left anterior insula that distinguished cohorts, with HC exhibiting greater values than SZ (Fig. 5d, Fig. 2 and Table 1). The robustness of our voxel-wise *t-*test assessment of cohort differences was confirmed by permutation test results from LIN (*ρ* > 0.999) and ENL (*ρ* > 0.999) pDM comparisons. Results for SUB, CER, VIS1, VIS2, MTR1, ATN, rFP and unique LIN network cohort comparisons are depicted in Supplementary Fig. 2. Summary cohort test information is provided in Supplementary Table 2 and summary sensitivity test information in Supplementary Table 3.

To validate the detection of SZ alterations within TEMP, MTR2, pDM and unique ENL networks, we used a genetic matching algorithm^55^ to balance HC and SZ cohorts for confounding factors and we subsequently analyzed networks derived from the balanced cohorts (detailed methods are provided in Supplementary Note 2). The matched analysis revealed a greater number of significant ENL voxels relative to LIN (Supplementary Fig. 3 and Supplementary Table 4) and validated our primary findings, strongly indicating that ENL estimates of the networks in question outperform LIN in capturing SZ FC alterations (summary test information is provided in Supplementary Table 4).

## Discussion

Linear FC analysis remains a fruitful method for extracting valuable information from fMRI data. However, despite its usefulness and ease of interpretation, various brain processes exhibit nonlinear aspects^15,17^, suggesting that linear FC provides us with a limited view of the data and the clinical neurocognitive hypothesis space. Previous rsfMRI studies have identified evidence of nonlinearity and its prospective role in differentiating cohorts^37,41,42^, but our approach to ENL ICN estimation demonstrates the potential of connectivity-domain ICA^33^ and nonlinear information to shape the predictive clinical landscape and inform systems neuroscience theorizing.

We find that components extracted from ENL-wFC exhibit higher reliability than those extracted from LIN-wFC, and that unique networks are identified from each FC estimator. Our validation analysis supports these findings. We also find that corresponding networks exhibit striking spatial variation. Among potential explanations, the presence of greater ENL weight within core regions could be reflective of stronger signals within core areas. However, we note that such a hypothesis probably cannot explain the detection of differences between the HC and SZ cohorts. Because ENL ICNs represent independent data sources composed of elements whose distance correlation values deviate from a linear relationship with Pearson correlation, the identified gradients may reflect actual differences in the underlying FC complexity, which merits further investigation of their potential cognitive and clinical significance. Notably, higher ENL weight within core regions indicates that linear connectivity analyses may underestimate FC within network centers. Future work will investigate potential explanations for the observed gradients.

The recovery of a unique ENL network underscores the importance of effectively capturing networks that accurately reflect nonlinear connectivity information, as our results show that networks estimated from information not explained by linear connectivity may be altered in psychiatric conditions such as SZ. For instance, the unique ENL network consolidated regions typically associated with cingulo-opercular^56^, anterior default mode^57^ and central executive^58^ networks, suggesting that this ICN may act as an integrator hub for multiple large-scale brain ensembles. This hypothesis is consistent with mounting evidence of a role for anterior insular regions in mediating information flow between default mode and central executive regions^59–61^. Moreover, anterior insular regions are associated with event and stimulus salience processing, both of which are reported to be compromised in SZ^62^. Importantly, group comparisons revealed functional hypoconnectivity for SZ within the left anterior insula of the unique ENL ICN, suggesting that our method can capture hidden patterns that reflect inefficiencies in the integration of brain networks in psychosis. This finding serves as a case in point connecting our methodology to the generation of novel insights, and demonstrates the potential of our approach to contribute to the development of brain-based biomarkers of psychiatric disorders.

The finding that ENL voxels collectively exhibit a greater degree of sensitivity to SZ diagnosis further elaborates the potential of nonlinear connectivity information to play a role within clinical FC analysis. ENL TEMP comparisons revealed larger clusters of significant voxels within auditory and language-related regions that have been previously associated with SZ and positive symptoms such as auditory verbal hallucinations in both tfMRI^63,64^ and rsfMRI^65,66^ analyses. For example, ENL tests revealed expansive clusters within superior temporal regions known to implement acoustic–phonetic computations^52^. ENL tests also identified a sizable volume with higher SZ values within the right supramarginal gyrus, which has been shown to play a role in phonological decision-making^67^. By contrast, the right supramarginal gyrus was almost entirely missed for significance by LIN TEMP comparisons. Notably, ENL MTR2 comparisons revealed greater numbers of significant voxels within sensorimotor regions previously implicated in SZ^66,68^, as well as clusters within the bilateral posterior insula that were not detected by LIN tests. Moreover, ENL pDM comparisons revealed hyperconnectivity for SZ that was missed by LIN within core regions of the pDM that have been associated with reflective, internally focused cognitive processes thought to be relevant to SZ diagnosis and symptoms^69^. This finding was validated by the matched cohort analysis (Supplementary Fig. 3). Overall, our results demonstrate that nonlinear statistical dependencies in fMRI data can be leveraged to distinguish these cohorts and warrant further investigation of the relationship between features extracted from measures that are sensitive to nonlinearity and the presentation of psychosis.

Our previous work proposed this conceptual framework^39^. Here, we advance and rigorously investigate the framework by providing an in-depth quantitative analysis of ENL and LIN networks, their spatial variation and their sensitivity to differences between HC and SZ. However, the current analysis has several methodological and interpretive limitations. First, although we utilized a large dataset collected across multiple psychosis projects and sites to address representative sampling issues^45^, the generalizability of our results is limited to populations of individuals with demographic characteristics similar to that of the analyzed sample. For instance, reporting on race for the present study utilized three super-population groupings (mixed American, European and African), and our results cannot necessarily be generalized to populations that fall outside these groups. Second, we note that alternate models of the relationship between NL-wFC and LIN-wFC can be leveraged when estimating ENL-wFC. Therefore, we do not claim that the current method of estimation is decisive or definitive to the potential exclusion of methods designed to estimate ENL-wFC using alternate models. Future work will investigate the use of other models with the aim of providing increasingly robust and precise characterizations of whole-brain connectivity features not explained by linear connectivity patterns. Third, we note that although our approach may share conceptual similarities with methods that construct nonlinear fMRI connectivity using features derived from pairwise associations^41,42^, we do not necessarily expect the findings of these distinct approaches to converge due to substantial differences in methodology. Thus, we leave any speculation about the relationship between features extracted from these methods as an open empirical question for future investigation. Fourth, we note that attributing context-invariant functions to macroscopic brain networks may oversimplify their roles. The functions attributed in the present study are suggested as those among the most supported by previous research findings. Finally, although our results warrant further investigation into the potential neurocognitive and psychiatric roles of ENL networks, we maintain that moving beyond association will probably require developing interventions that can effectively tie the extracted features to the causal outcomes of cognitive operations, psychiatric diagnosis and symptoms.

The primary goal of the current study was to investigate the presence and clinical utility of nonlinear FC patterns that move beyond linear FC. However, future work will investigate networks extracted from nonlinear FC patterns in the context of task-based experimental designs. Additionally, future work will focus on replicating our results in large-scale B-SNIP transdiagnostic rsfMRI datasets^70,71^, on utilizing ENL networks to distinguish a broader array of clinical cohorts, on analyzing associations with cognitive and symptom scores, and on analyzing the temporal^72^ and spatial^32,40,66,73,74^ dynamics exhibited by NL and ENL networks during task performance and at rest.

## Methods

### Subject information, data acquisition and quality control

We analyzed 3-Tesla rsfMRI data sourced from three case–control psychosis projects—COBRE, FBIRN and MPRC (Fig. 2 and Table 1). Detailed subject recruitment information, as well as inclusion and exclusion criteria for COBRE, FBIRN and MPRC studies, can respectively be found in refs. ^43,44,46^ as well as in the Reporting Summary. Sex was based on self-reported demographic assessment. Race was based on a multi-dimensional scaling (MDS) analysis conducted on combined local samples and data from the the 1000 Genomes Project^75^. For each super-population of mixed American (AMR), European (EUR) and African (AFR) individuals, a cluster centroid was obtained based on 1000 Genomes data. Local samples were assigned to the nearest reference population, and those that were distant (>3 s.d. away) from any population cluster were assigned to the ‘other’ category. Subjects provided informed written consent as required and approved by the Institutional Review Boards (IRBs) of the corresponding institutions. COBRE participants gave written informed consent as required and approved by the IRB of the University of New Mexico^43^. FBIRN participants gave written informed consent as required and approved by the IRBs of the University of California Irvine, the University of California Los Angeles, the University of California San Francisco, Duke University, University of North Carolina, University of New Mexico, University of Iowa and University of Minnesota^44^. MPRC participants gave written informed consent as required and approved by the IRB of the University of Maryland, Baltimore^46^.

Individuals with SZ from the COBRE dataset received a diagnosis of schizophrenia performed by two research psychiatrists in consensus via the Structured Clinical Interview for DSM-IV Axis I Disorders (SCID) using the patient version of the SCID-DSM-IV-TR^43^. SZ subjects were evaluated for comorbidities and for retrospective as well as prospective clinical stability. Individuals with SZ from the FBIRN study were diagnosed with schizophrenia based on the SCID-DSM-IV-TR and were clinically stable for at least two months before scanning^44^. For MPRC SZ subjects, a diagnosis of schizophrenia was confirmed via the SCID-DSM-IV^46^. Case and control participants were compensated for interviews, scan sessions and assessments conducted during the referenced studies.

COBRE data were collected at a single site on a Siemens TIM Trio scanner via an echo-planar imaging sequence (repetition time (TR) = 2,000 ms; echo time (TE) = 29 ms)^20^. Voxel spacing was 3.75 × 3.75 × 4.5 mm, the slice gap was 1.05 mm, and the field of view (FOV) was 240 × 240 mm. FBIRN data were collected from seven sites^76^, with six sites utilizing Siemens TIM Trio scanners and one utilizing a General Electric Discovery MR750 system^20^. All seven sites used an echo-planar imaging sequence (TR = 2,000 ms; TE = 30 ms). The original voxel spacing was 3.4375 × 3.4375 × 4 mm, the slice gap was 1 mm, and the FOV was 220 × 220 mm. MPRC data were collected from three sites via echo-planar imaging sequences^20^. One site used a Siemens Allegra scanner (TR = 2,000 ms; TE = 27 ms; voxel spacing = 3.44 × 3.44 × 4 mm; FOV = 220 × 220 mm), another used a Siemens TIM Trio scanner (TR = 2,210 ms; TE = 30 ms; voxel spacing = 3.44 × 3.44 × 4 mm; FOV = 220 × 220 mm), and the third site used a Siemens TIM Trio scanner (TR = 2,000 ms; TE = 30 ms; voxel spacing = 1.72 × 1.72 × 4 mm; FOV = 220 × 220 mm).

The following subject quality control criteria^21^ were used for the current study: (1) completeness of demographic information, (2) availability of T1 structural MRI, (3) availability of genomic information, (4) maximum head rotation less than 3°, (5) maximum translation less than 3 mm, (6) mean framewise displacement less than 0.25, (7) quality registration to an echo-planar imaging template, (8) whole-brain (in addition to the top ten and bottom ten slices) spatial overlap between the subject mask and group mask greater than 80% and (9) removal of duplicate subjects. The final subject pool included 315 HC and 193 SZ (*n* = 508) individuals.

### Preprocessing

Preprocessing was performed primarily within the MATLAB software environment using Statistical Parametric Mapping (SPM 12; http://www.fil.ion.ucl.ac.uk/spm/) and the FMRIB Software Library (FSL v6.0; https://fsl.fmrib.ox.ac.uk/fsl/fslwiki). Preprocessing steps included (1) rigid body motion and slice timing correction, (2) nonlinear warping to the Montreal Neurological Institute 152 coordinate space, (3) spatial resampling to 3-mm isotropic voxel spacing, (4) spatial smoothing with a 6-mm full-width at half-maximum Gaussian kernel, (5) head motion regression, detrending, despiking and low-pass filtering, (6) temporal resampling to TR = 2,000 ms and (7) voxel time-series *Z*-scoring to normalize the variance.

### Constructing LIN and ENL FC

We constructed LIN as well as ENL global (voxel-wise) FC matrices for every subject^39^. Let ${X\in {{\mathbb{R}}}^{n\times v}}$ be a sample of rsfMRI data where *n* is the number of time points, *v* is the number of voxels within the brain, and *x* and *y* represent any two preprocessed voxel time series such that $x,\,y\,{\in \,{{\mathbb{R}}}^{1\times n}}$. Thus, *x*_*i*_ is the value of voxel *x* at time point *i*. We estimated each subject’s LIN-wFC as the covariance (Cov) across all pairs of brain voxels (equation (1)). Because voxel time courses were *Z*-scored during preprocessing, the pairwise covariance was equal to the pairwise Pearson correlation, which was used conventionally to estimate linear FC:

1 $${\rm{LIN}}_{{\rm{wFC}}_{x,\,y}}={{\rm{Cov}}\left(x,\,y\right)=\frac{1}{n-1}\mathop{\sum }\limits_{i=1}^{n}\left({x}_{i}\right)\left(\,{y}_{i}\right)}$$

Next, we calculated the voxel-wise distance correlation^38^ to construct NL-wFC. Distance correlation is a representation of the association between random vectors based on Euclidean distances between sample observations^38^ (equation (2)):

2 $${\rm{NL}}_{{\rm{wFC}}_{x,\,y}}={{\rm{dCorr}}\left(x,\,y\right)={\frac{{\rm{dCov}}\left(x,\,y\right)}{\sqrt{{\rm{dVar}}\left(x\right){\rm{dVar}}\left(\,y\right)}}}}$$

where

$${{\rm{dCov}}_{n}^{2}\left(x,\,y\right)}={\frac{1}{{n}^{2}}\mathop{\sum }\limits_{j=1}^{n}\mathop{\sum }\limits_{k=1}^{n}{A}_{j,\,k}{B}_{j,\,k}}$$

and

$${{\rm{dVar}}_{n}^{2}\left(x\right)}={{\rm{dCov}}_{n}^{2}(x,\,x)}={\frac{1}{{n}^{2}}\mathop{\sum }\limits_{k=1}^{n}\mathop{\sum }\limits_{l=1}^{n}{A}_{k,\,l}^{2}}$$

The squared sample distance covariance (dCov^2^) is calculated as the arithmetic average of products *AB*, where *A* and *B* represent the doubly centered Euclidean distance matrices of rsfMRI voxel time series *x* and *y* such that

$${a}_{j,\,k}={\Vert {x}_{j}-{x}_{k}\Vert \,\,\,j,\,k=1,\,2,\,\ldots ,\,n}$$

$${b}_{j,\,k}={\Vert\,{y}_{j}-{y}_{k}\Vert ,\,\,\,j,\,k=1,\,2,\ldots ,\,n}$$

$${A}_{j,\,k}={a}_{j,\,k}-{\bar{a}}_{j\cdot }-{\bar{a}}_{\cdot k}-{\bar{a}}_{\cdot \cdot },$$

$${B}_{j,\,k}={b}_{j,\,k}-{\bar{b}}_{j\cdot }-{\bar{b}}_{\cdot k}-{\bar{b}}_{\cdot \cdot }$$

We note that distance correlation is sensitive to both linear and nonlinear dependence relations, and that the distance correlation between random vectors is zero if and only if the vectors are independent^38^.

Because we are interested in extracting networks from distance correlation patterns that are not explained by Pearson correlation, we removed the effect of LIN-wFC on NL-wFC using an ordinary least-squares approach to estimate the ENL-wFC for each subject (equation (3)). We first vectorized both NL-wFC and LIN-wFC. We then removed the linear relationship between NL-wFC and LIN-wFC using a regression-based method and reshaped the vector of residuals into a *v* × *v* FC matrix:

3 $${\rm{ENL}}_{\rm{wFC}}={\rm{vec}}^{-1}\left({\rm{vec}}\left({\rm{NL}}_{\rm{wFC}}\right)-\alpha \times {\rm{vec}}\left({\rm{LIN}}_{\rm{wFC}}\right)\right)$$

where

$$\mathop{{min }}\limits_{\alpha }\mathop{\sum }\limits_{i=1}^{{v}^{2}}({{\left({\rm{vec}}\left({\rm{NL}}_{\rm{wFC}}\right)\right)}_{i}-{\left({\rm{vec}}\left({\rm{LIN}}_{\rm{wFC}}\right)\right)}_{i}})^{2}$$

We treated the estimation of *α* as an ordinary least-squares problem by finding the value of *α* that minimized the sum of squared errors between NL-wFC and LIN-wFC. Thus, here we define the ENL-wFC for a given subject as the NL-wFC information with the linear effect of LIN-wFC removed. For each subject, the goodness of fit of the linear model was evaluated via the coefficient of determination (*R*^2^). To assess the difference in *R*^2^ between HC and SZ cohorts, we used a general linear model (GLM) to remove the effect of confounding factors commonly reported in psychosis studies, including age, sex, site and motion (mean framewise displacement), on the goodness-of-fit data, and we subsequently conducted a two-sided permutation test with 5,000 random permutations (Krol, 2023; https://github.com/lrkrol/permutationTest)^77^.

### Extracting ICNs

We used the Group ICA of the fMRI Toolbox (GIFT v4.0; http://trendscenter.org/software/gift)^72^ to implement connectivity-domain ICA^33^ and obtain separate sets of group-level networks from the LIN-wFC and ENL-wFC data. The implementation of gr-sICA was preceded by an initial subject-level, multi-power iteration^78^, principal component analysis step to reduce dimensionality and denoise the data^79^. The 30 principal components that explained the maximum variance of each subject’s respective LIN-wFC and ENL-wFC were retained for further analysis. Subject-level principal components from each estimator were concatenated across the component dimension, and a group-level principal component analysis step was applied to further reduce the dimensionality of the data and decrease the computational demands of gr-sICA^11^. The 20 group-level principal components that explained the maximum variance of each estimator-specific dataset were used as the input for gr-sICA. We selected a gr-sICA model order of 20 to obtain large-scale functional networks^33,80^. To ensure the reliability of our results, ICA was implemented via the Infomax optimization algorithm^81^ 100 times, with both random initialization and bootstrapping, and the most stable run was selected for further analysis. We evaluated the reliability and quality of ENL and LIN components using the ICASSO quality index (IQ), which quantifies component stability across runs^82^. To assess the difference in stability between ENL and LIN components, we conducted a two-sided permutation test with 5,000 random permutations on the IQ data. Assessing component reliability was a necessary step, as previous work has demonstrated that certain components may be inconsistently extracted from the data of interest^82^. In the context of fMRI network estimation, ICASSO IQ is often used to differentiate reliable components from components that are unstable and unfit for further analysis^66^. A component was identified as an ICN if and only if (1) it exhibited an ICASSO IQ value exceeding 0.80, (2) it exhibited high visual overlap with gray matter, (3) it exhibited peak weight within gray matter, and (4) it exhibited low visual similarity to motion, ventricular and other known artefacts. To find corresponding networks, the spatial correlation value was computed between every pair of extracted LIN and ENL components, and components were matched in a greedy fashion. ICNs matched with a spatial correlation value exceeding 0.80 were classified as common^21^ and were labeled based on their neuroanatomical distributions and the identification of ICNs from previous studies^33^. Networks exhibiting a maximum spatial correlation of less than 0.40 were classified as unique. We used the Group ICA of fMRI Toolbox (GIFT v4.0) to implement group information-guided ICA (GIG-ICA)^83^ and reconstruct subject-specific networks from subject-level principal components using the group-level spatial references.

### Assessment of spatial variation among corresponding ICNs

To assess differences in spatial variation between matched networks, we conducted voxel-wise, two-sided, paired-samples *t*-tests on their *Z*-scored subject-level estimates. For a given matched network pair, statistical comparisons were masked for voxels exceeding *Z* = 1.96 (*P* = 0.05) in either group-level map (LIN or ENL), and the false discovery rate (FDR)^84^ method was used to correct for multiple comparisons (*q* < 0.05). The robustness of the voxel-wise *t*-test procedure was assessed via comparison to the results of voxel-wise two-sided permutation tests with 5,000 random permutations for the pDM network. The automated anatomical labeling atlas 3 (AAL3)^85^ was used to localize clusters of significant voxels to anatomically defined brain regions.

### Assessment of ICN differences between HC and SZ

To assess the differences between HC and SZ, we conducted voxel-wise, independent-samples *t*-tests between the estimates of common and unique networks derived from each cohort. We first used a GLM to remove the effects of confounding factors such as age, sex, site and motion (mean framewise displacement) on *Z*-scored subject-level network estimates. Voxel-wise, two-sided, independent-samples *t*-tests were then conducted on the residual spatial maps derived from the HC and SZ groups. Statistical comparisons between common networks were masked for voxels exceeding *Z* = 1.96 (*P* = 0.05) in either of the group-level maps (LIN or ENL), and unique network comparisons were masked for voxels exceeding the same threshold in the unique group-level map. The FDR^84^ method was used to correct for multiple comparisons (*q* < 0.05). For both ENL and LIN, the robustness of the voxel-wise *t*-test procedure was assessed via comparison to the results of voxel-wise, two-sided permutation tests with 5,000 random permutations for the pDM network. The AAL3^85^ atlas was used to localize clusters of significant voxels to anatomically defined brain regions. A two-sided McNemar’s test was used to assess the overall ENL versus LIN difference in statistical sensitivity (across all voxels belonging to commonly classified networks), and differences in statistical sensitivity for matched network pairs were investigated separately using either two-sided McNemar’s tests or exact binomial tests (for *n* < 25).

### Reporting summary

Further information on research design is available in the Nature Portfolio Reporting Summary linked to this Article.

## Supplementary information

Supplementary Information Supplementary Notes 1 and 2, Figs. 1–3 and Tables 1–4.

Reporting Summary

## Data Availability

Contact information and resources for obtaining further details for the private datasets utilized in the present study are as follows. COBRE: Vince D. Calhoun (vcalhoun@gsu.edu), Tri-Institutional Center for Translational Research in Neuroimaging and Data Science (TReNDS), Atlanta, GA, USA^43^. FBIRN: Theo G. M. van Erp (tvanerp@hs.uci.edu), Clinical Translational Neuroscience Laboratory, Department of Psychiatry and Human Behavior, University of California, Irvine, CA, USA^45^. MPRC: Peter Kochunov (ms.psychiatry@uth.tmc.edu), Department of Psychiatry and Behavioral Science, University of Texas Health Science Center Houston, Houston, TX^46^.

## Code availability

Preprocessing and data analysis were conducted primarily within the MATLAB software environment mainly using MATLAB 9.9.0.1857802 (R2020b) Update 7, the Statistical Parametric Mapping toolbox (SPM 12), the FMRIB software library (FSL v6.0), the Group ICA of fMRI toolbox (GIFT v4.0) and RStudio (R v4.1.2). MATLAB R2020b can be downloaded from https://www.mathworks.com. The FSL v6.0 toolbox can be downloaded from https://fsl.fmrib.ox.ac.uk/fsl/fslwiki. The SPM 12 toolbox can be downloaded from https://www.fil.ion.ucl.ac.uk/spm/. GIFT v4.0 can be downloaded from https://trendscenter.org/software/gift/. R v4.1.2 can be downloaded from https://cran.r-project.org/. The sample scripts utilized for dual code data visualization^86^ can be downloaded from https://trendscenter.org/x/datavis/. The permutation test function utilized for statistical randomization analyses (Krol, 2023)^77^ can be downloaded from https://github.com/lrkrol/permutationTest/. The R package used to balance HC and SZ cohorts for confounding factors and assess differences using Abadie–Imbens standard errors^87^ can be downloaded from https://CRAN.R-project.org/package=Matching. The MATLAB function used to calculate ENL-wFC can be downloaded from https://github.com/trendscenter/calc_ENLwFC. Other MATLAB code used for this study can be obtained from the corresponding authors upon reasonable request.
