# Supplementary material for: Networks extracted from nonlinear fMRI connectivity exhibit unique spatial variation and enhanced sensitivity to differences between individuals with schizophrenia and controls
Source: Nat Ment Health. 2024 Nov 21;2(12):1464–75. doi: 10.1038/s44220-024-00341-y (PMC11621020; doi:10.1038/s44220-024-00341-y)
Supplement: Supplementary file 1 — Supplementary Notes 1 and 2, Figs. 1–3 and Tables 1–4. [file 44220_2024_341_MOESM1_ESM.pdf]

nature mental health

Supplementary information https://doi.org/10.1038/s44220-024-00341-y

Networks extracted from nonlinear fMRI connectivity exhibit unique spatial variation and enhanced sensitivity to differences between individuals with schizophrenia and controls

In the format provided by the authors and unedited

Supplementary Information

Supplementary Note 1

ENL ICN Uniqueness: Validation

Although the spatial distribution of a component extracted from linear whole-brain functional connectivity (LIN-wFC) group-level spatial ICA (gr-sICA) showed similarity of .8933 to the explicitly nonlinear (ENL) network that we classified as unique (Fig. 2), the linear (LIN) component in question was not reliably estimated, exhibiting an ICASSO quality index (IQ) value of .6186, which fell below our estimation reliability threshold (.80)[66]. Previous research supports the view that this strongly suggests the component is inconsistently extracted from the LIN-wFC data and unfit to be analyzed as a LIN intrinsic connectivity network (ICN) despite the similarity of its spatial distribution[66,82]. To validate this result, we conducted 100 additional iterations of gr-sICA on the LIN-wFC and ENL whole-brain functional connectivity (ENL-wFC) data. For each additional iteration, a randomized subset of subjects comprising 80% of the total subject pool was selected for analysis. Gr-sICA parameters were identical to those of the full analysis except for the number of Infomax runs, which was equal to five. The components extracted during each iteration were matched based on spatial correlation with ENL components extracted from the full analysis. Using a spatial similarity threshold of .80 and our ICN inclusion criteria, we determined that the ICN of interest was identified in 78/100 of the additional ENL-wFC analyses (Supplementary Fig. 1a) while it was identified in only 9/100 of the additional LIN-wFC analyses (Supplementary Fig. 1b). This result indicates that the network in question cannot be reliably estimated from LIN-wFC data within a 20-model-order gr-sICA framework.

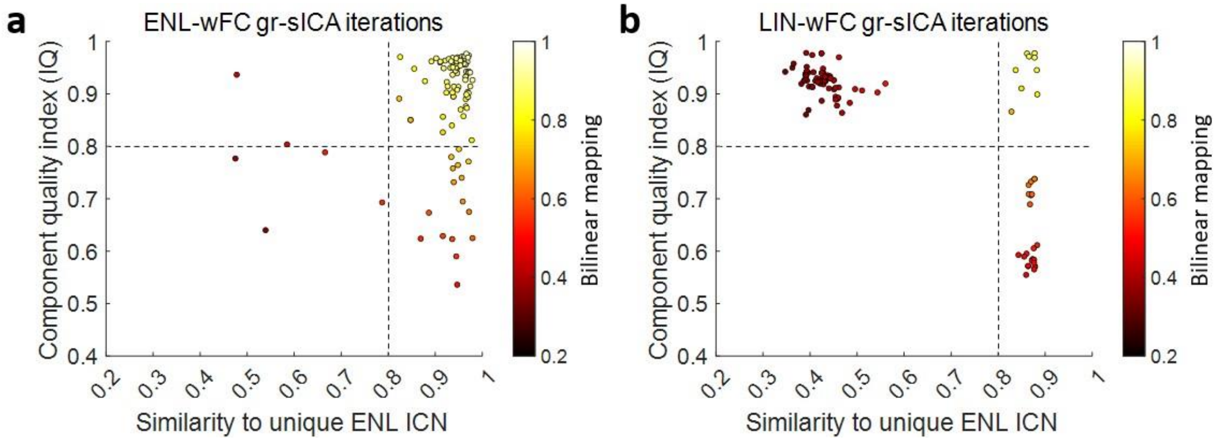

Supplementary Fig. 1: Scatterplots of explicitly nonlinear whole-brain functional connectivity (ENL-wFC) and linear whole-brain functional connectivity (LIN-wFC) group-level spatial independent component analysis (gr-

sICA) iterations. Each iteration is plotted according to a colormap reflecting the bilinear mapping between the spatial correlation of the component matched with the unique ENL ICN and that component's ICASSO quality index (IQ) value. The broken lines demarcate spatial similarity and IQ thresholds of .80. ENL-wFC gr-sICA iterations (a) cluster within the top right quadrant of the plot, indicating that matched components extracted from ENL-wFC analyses generally exhibited suprathreshold spatial similarity and suprathreshold ICASSO IQ values. This pattern is not observed in the LIN-wFC plot (b), which reveals that most LIN-wFC gr-sICA iterations failed to identify the unique ENL ICN.

Supplementary Table 1 Summary information for voxel-wise statistical comparisons between corresponding explicitly nonlinear (ENL) and linear (LIN) intrinsic connectivity network (ICN) estimates. Statistical tests were two-sided paired samples *t*-tests. Summary confidence interval (CI) information for all tested voxels is reported as median, minimum, and maximum interval range. Summary standardized effect size information for all significant voxels is reported as median, minimum, and maximum mean difference divided by standard error (Cohen's *d*). SUB: subcortical. CER: cerebellum. VIS1: primary visual. VIS2: secondary visual. TEMP: temporal. MTR1: primary sensorimotor. MTR2: secondary sensorimotor. ATN: dorsal attention. pDM: posterior default mode. rFP: right frontoparietal.

**ICN**	***n***	***df***	**CI range median / min - max**	**Cohen's *d* median / min - max**
SUB	508	507	0.1885 / 0.1404 - 0.4228	0.3279 / 0.0903 - 0.7847
CER	508	507	0.1970 / 0.1402 - 0.3500	0.2958 / 0.0903 - 0.7135
VIS1	508	507	0.1495 / 0.1175 - 0.2170	0.2572/ 0.0927 - 0.8253
VIS2	508	507	0.1682 / 0.1279 - 0.2426	0.1976 / 0.0969 - 0.5131
TEMP	508	507	0.1740 / 0.1351 - 0.2295	0.3676 / 0.0906 - 1.0761
MTR1	508	507	0.1482 / 0.1129 - 0.2030	0.2350 / 0.0940 - 0.7134
MTR2	508	507	0.1636 / 0.1281 - 0.2405	0.2316 / 0.0929 - 0.7062
ATN	508	507	0.1663 / 0.1222 - 0.2599	0.3618 / 0.0898 - 0.9228
pDM	508	507	0.1679 / 0.1216 - 0.2453	0.3761 / 0.0905 - 1.0974
rFP	508	507	0.1799 / 0.1329 - 0.3058	0.2531 / 0.0911 - 0.8943

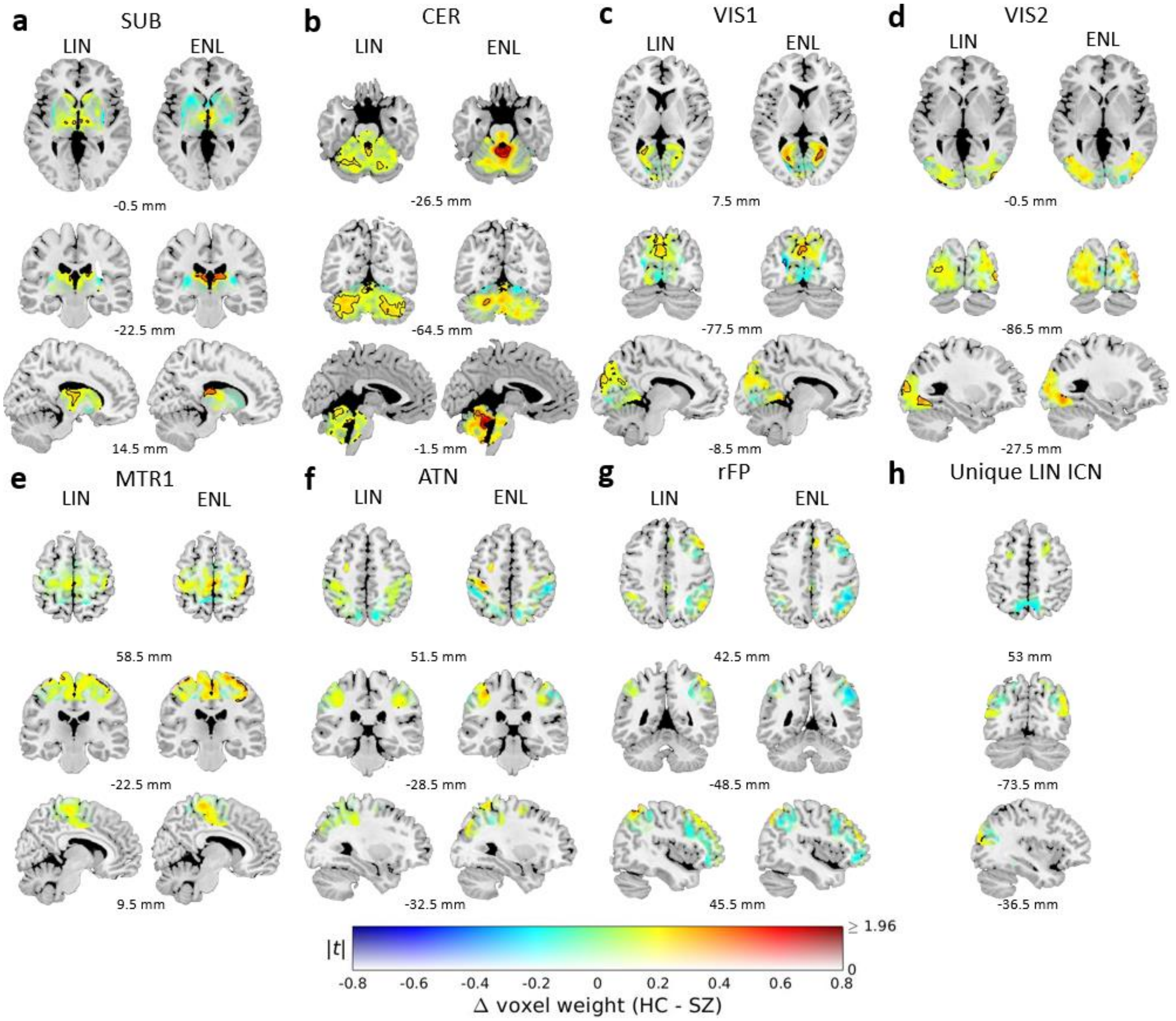

Supplementary Fig. 2: Statistical comparisons between subject-level subcortical (SUB) (a), cerebellum (CER) (b), primary visual (VIS1) (c), secondary visual (VIS2) (d), primary sensorimotor (MTR1) (e), dorsal attention (ATN) (f), right frontoparietal (rFP) (g), and unique linear (LIN) (h) intrinsic connectivity network (ICN) estimates derived from healthy control (HC) and schizophrenia (SZ) cohorts. Results are plotted according to a dual coded[86] colormap with transparency reflecting two-sided paired samples *t* statistic magnitudes and contours indicating FDR corrected statistical significance ($q < .05$). Warmer hues indicate HC > SZ, while cooler hues indicate SZ > HC. Results are overlaid on the ch2bet template with X, Y, and Z coordinates listed relative to the origin in Montreal Neurological Institute 152 space.

Supplementary Table 2 Summary information for voxel-wise statistical comparisons between explicitly nonlinear (ENL) and linear (LIN) intrinsic connectivity network (ICN) estimates derived from healthy control (HC) and schizophrenia (SZ) cohorts. Statistical tests were two-sided independent samples *t*-tests. Summary confidence interval (CI) information for all tested voxels is reported as median, minimum, and maximum interval range. Summary standardized effect size information for all significant voxels is reported as median, minimum, and maximum mean difference divided by standard error (Cohen's *d*). SUB: subcortical. CER: cerebellum. VIS1: primary visual. VIS2: secondary visual. TEMP: temporal. MTR1: primary sensorimotor. MTR2: secondary sensorimotor. ATN: dorsal attention. pDM: posterior default mode. rFP: right frontoparietal.

ICN	Number of significant voxels (ENL)	Number of significant voxels (LIN)	*n*	*df*	CI range median / min - max (ENL)	CI range median / min - max (LIN)	Cohen's *d* median / min - max (ENL)	Cohen's *d* median / min - max (LIN)
SUB	131	141	508	506	0.4001 / 0.2926 - 0.6281	0.3157 / 0.2674 - 0.4220	0.3213 / 0.2859 - 0.4483	0.3177 / 0.2838 - 0.4885
CER	424	1184	508	506	0.4148 / 0.3064 - 0.7606	0.3076 / 0.2605 - 0.3940	0.2963 / 0.2642 - 0.4327	0.2753 / 0.2335 - 0.4763
VIS1	361	558	508	506	0.4248 / 0.3434 - 0.5573	0.2913 / 0.2264 - 0.4410	0.2960 / 0.2603 - 0.4087	0.2856 / 0.2470 - 0.4509
VIS2	12	405	508	506	0.4313 / 0.3457 - 0.5515	0.3112 / 0.2640 - 0.4054	0.3576 / 0.3507 - 0.3800	0.3034 / 0.2582 - 0.4853
TEMP	1236	306	508	506	0.3882 / 0.2927 - 0.4855	0.3114 / 0.2471 - 0.3826	0.3108 / 0.2280 - 0.6228	0.3100 / 0.2709 - 0.4254
MTR1	59	84	508	506	0.3874 / 0.3073 - 0.5301	0.2831 / 0.2219 - 0.3888	0.3332 / 0.3130 - 0.4209	0.3342 / 0.3032 - 0.4323
MTR2	1513	680	508	506	0.4030 / 0.3068 - 0.5623	0.2771 / 0.2327 - 0.3841	0.2844 / 0.2187 - 0.5158	0.2992 / 0.2446 - 0.5471
ATN	0	0	508	506	0.4187 / 0.2972 - 0.5121	0.2933 / 0.2326 - 0.4025	-	-
pDM	129	2	508	506	0.4120 / 0.2592 - 0.5328	0.2884 / 0.2201 - 0.3835	0.3246 / 0.2906 - 0.4200	0.3975 / 0.3919 - 0.4030
rFP	0	12	508	506	0.4562 / 0.3214 - 0.7239	0.3307 / 0.2582 - 0.4943	-	0.3850 / 0.3516 - 0.4130
Unique LIN	-	0	508	506	-	0.3176 / 0.2524 - 0.4070	-	-
Unique ENL	60	-	508	506	0.4051 / 0.3116 - 0.5863	-	0.3272 / 0.3088 - 0.4334	-
Total	3925	3372		-	-	-	-	-

Supplementary Table 3 Results from statistical sensitivity testing between explicitly nonlinear (ENL) and linear (LIN) intrinsic connectivity network (ICN) estimates derived from healthy control (HC) and schizophrenia (SZ) cohorts. A two-sided McNemar's test was used to assess the overall ENL vs. LIN difference in statistical sensitivity (across all voxels belonging to commonly classified networks), and differences in statistical sensitivity for matched network pairs were assessed separately using either two-sided McNemar's tests or exact binomial tests (for $n < 25$). Odds ratio (OR) is used as an indicator of effect size. SUB: subcortical. CER: cerebellum. VIS1: primary visual. VIS2: secondary visual. TEMP: temporal. MTR1: primary sensorimotor. MTR2: secondary sensorimotor. ATN: dorsal attention. pDM: posterior default mode. rFP: right frontoparietal.

ICN	Number of significant voxels identified by ENL but not LIN	Number of significant voxels identified by LIN but not ENL	*df*	x^2	*p*	OR
SUB	101	111	1	0.4717	.4922	-
CER	256	1006	1	445.7	< .00001	3.93
VIS1	154	351	1	76.85	< .00001	2.28
VIS2	1	394	1	391	< .00001	394
TEMP	973	43	1	851.3	< .00001	22.63
MTR1	33	58	1	6.868	.0088	1.76
MTR2	959	126	1	639.5	< .00001	7.61
ATN	0	0	-	-	-	-
pDM	128	1	1	125.03	< .00001	128
rFP	0	12	1	-	< .00001	Undefined
Total	2605	2102	1	53.75	< .00001	1.24

Supplementary Note 2

Identifying Matched Cohorts via Optimal Covariate Balance

We used a multivariate genetic matching algorithm[55] to identify a subset of healthy controls (HC) and individuals with schizophrenia (SZ) that were optimally balanced for age, sex, site, and motion (mean framewise displacement). After matching with replacement, bootstrapped Kolmogorov-Smirnov statistics were computed for non-dichotomous covariates (age, site, and motion) over 5000 runs to confirm balance, while a two-sided paired samples t-test approach was used to assess balance for sex. Results confirmed that the matching process resulted in HC and SZ cohorts balanced for age (KS statistic = 0.0570; $p = .8174$), sex ($t = -0.1270$; $p = .8990$), site (KS statistic = 0.0363; $p = .9122$), and motion (KS statistic = 0.0466; $p = .9792$).

Assessment of Effect of SZ on ICN Voxel Weight

To validate the detection of SZ FC alterations by ENL and LIN methods using the balanced data, we estimated the voxel-wise bias-adjusted average treatment effect for the treated (ATT, where SZ diagnosis was considered the treatment factor) and Abadie-Imbens[87] standard error from the Z-scored subject-level estimates of temporal (TEMP), secondary sensorimotor (MTR2), posterior default mode (pDM), and unique ENL ICNs. We subsequently conducted two-sided Z-tests to evaluate effects for significance. Common ICN statistical comparisons were masked for voxels exceeding $Z = 1.96$ ($p = .05$) in either of the group-level maps (LIN or ENL), while unique ENL comparisons were masked for voxels exceeding the same threshold in the unique group-level map. The False Discovery Rate (FDR)[84] method was used to correct for multiple comparisons ($q < .05$). Results from the matched cohort analysis (Supplementary Fig. 3) validate our primary findings and strongly indicate that ENL estimates of the ICNs in question outperform LIN in the detection of SZ FC alterations. Summary test information is provided in Supplementary Table 4.

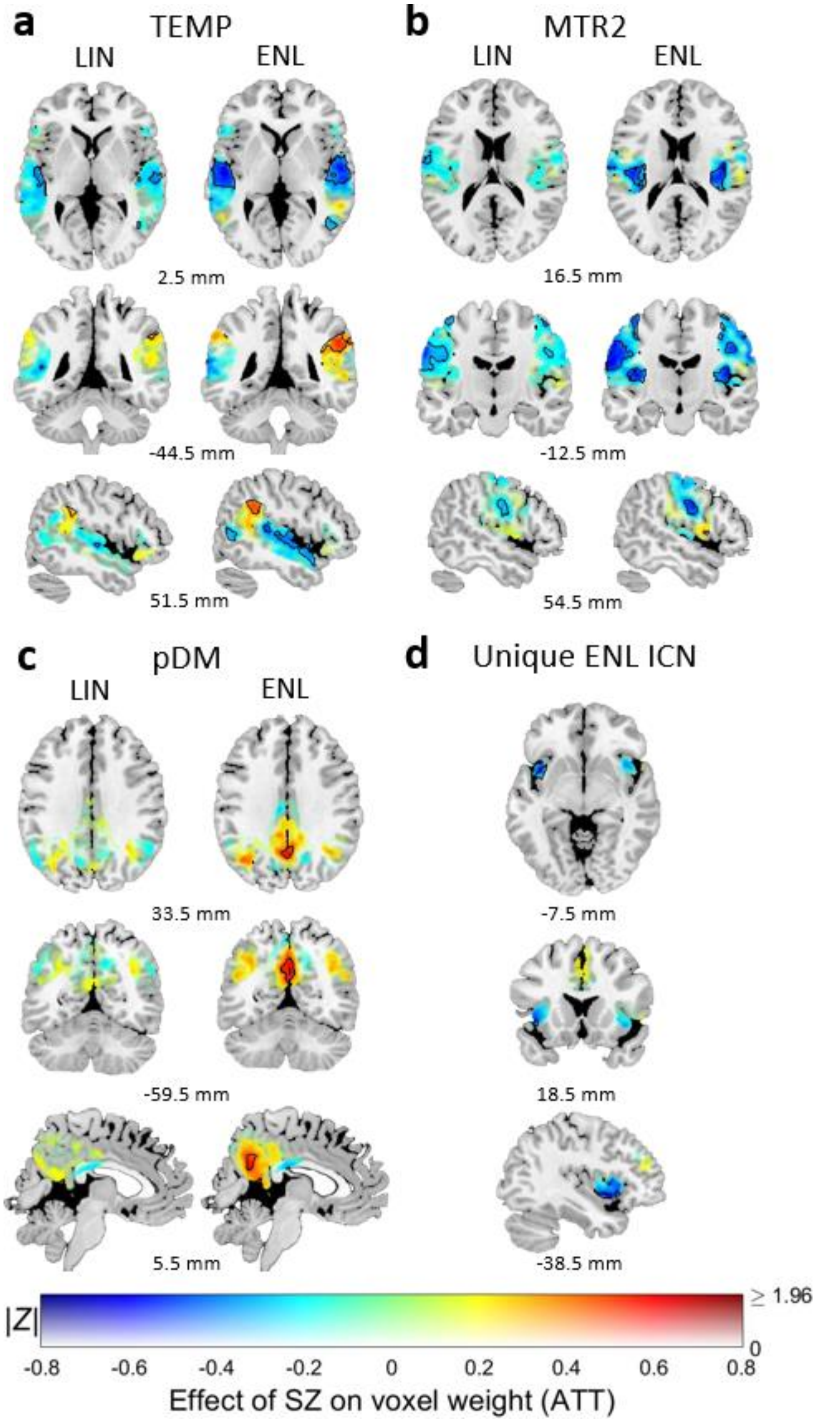

Supplementary Fig. 3: Statistical assessment of the effect of schizophrenia (SZ) diagnosis on explicitly nonlinear (ENL) and linear (LIN) subject-level temporal (TEMP) (a), secondary sensorimotor (MTR2) (b), posterior default mode (pDM) (c), and unique ENL (d) ICN estimates after balancing cohorts for age, sex, site, and motion (mean framewise displacement). Results are plotted according to a dual coded[86] colormap with transparency reflecting two-sided *Z* statistic magnitudes and contours indicating FDR-corrected statistical significance ($q < .05$). In a-c, results from LIN comparisons are located on the left, while results from ENL comparisons are located on the right. Warmer hues indicate a positive relationship between SZ diagnosis and voxel weight, while cooler hues

indicate a negative relationship. Results are overlaid on the ch2bet template with X, Y, and Z coordinates listed relative to the origin in Montreal Neurological Institute 152 space. ATT: average treatment effect for the treated.

Supplementary Table 4 Summary test information for voxel-wise effect of schizophrenia (SZ) diagnosis on explicitly nonlinear (ENL) and linear (LIN) intrinsic connectivity network (ICN) estimates after balancing cohorts for age, sex, site, and motion (mean framewise displacement). Statistical tests were two-sided *Z*-tests. Summary confidence interval (CI) information for all tested voxels is reported as median, minimum, and maximum interval range. Summary effect size information for all significant voxels is reported as median, minimum, and maximum average treatment effect for the treated (ATT) estimate. TEMP: temporal. MTR2: secondary sensorimotor. pDM: posterior default mode. HC: healthy control.

ICN	Number of significant voxels (ENL)	Number of significant voxels (LIN)	*n* excluding / including repeated HC observations	CI range median / min - max (ENL)	CI range median / min - max (LIN)	ATT estimate median / min - max (ENL)	ATT estimate median / min - max (LIN)
TEMP	808	261	319 / 386	0.5422 / 0.3609 - 0.7568	0.4340 / 0.3137 - 0.5694	-0.4254 / -0.8058 - 0.6557	-0.3499 / -0.5978 - 0.4570
MTR2	1020	585	319 / 386	0.5565 / 0.3814 - 0.8069	0.3884 / 0.2976 - 0.5546	-0.4562 / -0.9202 - 0.4843	-0.3348 / -0.6198 - 0.3130
pDM	112	0	319 / 386	0.5499 / 0.3476 - 0.8101	0.3984 / 0.2881 - 0.5648	0.5157 / -0.4927 - 0.6604	-
Unique ENL	16	-	319 / 386	0.5613 / 0.3845 - 0.8665	-	-0.5703 / -0.6589 - -0.3651	-
