# Supplementary material for: Networks extracted from nonlinear fMRI connectivity exhibit unique spatial variation and enhanced sensitivity to differences between individuals with schizophrenia and controls
Source: Nat Ment Health. 2024 Nov 21;2(12):1464–75. doi: 10.1038/s44220-024-00341-y (PMC11621020; doi:10.1038/s44220-024-00341-y)
Supplement: Supplementary file 2 — Reporting Summary [file 44220_2024_341_MOESM2_ESM.pdf]

nature portfolio

Corresponding author(s): Spencer Kinsey and Armin Iraji

Last updated by author(s): 08/02/2024

Reporting Summary

Nature Portfolio wishes to improve the reproducibility of the work that we publish. This form provides structure for consistency and transparency in reporting. For further information on Nature Portfolio policies, see our Editorial Policies and the Editorial Policy Checklist.

Statistics

For all statistical analyses, confirm that the following items are present in the figure legend, table legend, main text, or Methods section.

n/a	Confirmed	
☐	☒	The exact sample size (*n*) for each experimental group/condition, given as a discrete number and unit of measurement
☐	☒	A statement on whether measurements were taken from distinct samples or whether the same sample was measured repeatedly
☐	☒	The statistical test(s) used AND whether they are one- or two-sided<br>*Only common tests should be described solely by name; describe more complex techniques in the Methods section.*
☐	☒	A description of all covariates tested
☐	☒	A description of any assumptions or corrections, such as tests of normality and adjustment for multiple comparisons
☐	☒	A full description of the statistical parameters including central tendency (e.g. means) or other basic estimates (e.g. regression coefficient) AND variation (e.g. standard deviation) or associated estimates of uncertainty (e.g. confidence intervals)
☐	☒	For null hypothesis testing, the test statistic (e.g. *F*, *t*, *r*) with confidence intervals, effect sizes, degrees of freedom and *P* value noted<br>*Give P values as exact values whenever suitable.*
☒	☐	For Bayesian analysis, information on the choice of priors and Markov chain Monte Carlo settings
☒	☐	For hierarchical and complex designs, identification of the appropriate level for tests and full reporting of outcomes
☐	☒	Estimates of effect sizes (e.g. Cohen's *d*, Pearson's *r*), indicating how they were calculated

Our web collection on statistics for biologists contains articles on many of the points above.

Software and code

Policy information about availability of computer code

Data collection	No software was used for data collection. The present study was based on referenced datasets.
Data analysis	Preprocessing and data analysis were conducted primarily within the MATLAB software environment mainly using MATLAB 9.9.0.1857802 (R2020b) Update 7, the Statistical Parametric Mapping toolbox (SPM 12), the FMRIB software library (FSL v6.0), the Group ICA of fMRI toolbox (GIFT v4.0), and RStudio (R v4.1.2).<br><br>MATLAB R2020b can be downloaded from https://www.mathworks.com.<br><br>The FSL v6.0 toolbox can be downloaded from https://fsl.fmrib.ox.ac.uk/fsl/fslwiki.<br><br>The SPM 12 toolbox can be downloaded from https://www.fil.ion.ucl.ac.uk/spm/.<br><br>GIFT v4.0 can be downloaded from https://trendscenter.org/software/gift/.<br><br>R v4.1.2 can be downloaded from https://cran.r-project.org/.<br><br>The sample scripts utilized for dual code data visualization (Allen et al., 2012; https://doi.org/10.1016/j.neuron.2012.05.001) can be downloaded from https://trendscenter.org/x/datavis/.<br><br>The permutation test function utilized for statistical randomization analyses (Krol, 2023) can be downloaded from https://github.com/lrkrol/permutationTest/.

The R package used to balance healthy control (HC) and schizophrenia (SZ) cohorts for confounding factors can be downloaded from https://CRAN.R-project.org/package=Matching.

Other MATLAB code used for this study can be obtained from the corresponding authors.

For manuscripts utilizing custom algorithms or software that are central to the research but not yet described in published literature, software must be made available to editors and reviewers. We strongly encourage code deposition in a community repository (e.g. GitHub). See the Nature Portfolio guidelines for submitting code & software for further information.

Data

Policy information about availability of data

All manuscripts must include a data availability statement. This statement should provide the following information, where applicable:

- Accession codes, unique identifiers, or web links for publicly available datasets
- A description of any restrictions on data availability
- For clinical datasets or third party data, please ensure that the statement adheres to our policy

Contact information and resources for obtaining further details for the private datasets utilized in the present study are listed as follows:

COBRE: Vince D. Calhoun (vcalhoun@gsu.edu), Tri-Institutional Center for Translational Research in Neuroimaging and Data Science (TReNDS), Atlanta, GA, USA (Aine et al., 2017; https://doi.org/10.1007/s12021-017-9338-9)

FBIRN: Theo G. M. van Erp (tvanerp@hs.uci.edu), Clinical Translational Neuroscience Laboratory, Department of Psychiatry and Human Behavior, University of California, Irvine, CA, USA
(Keator et al., 2016; https://doi.org/10.1016/j.neuroimage.2015.09.003)

MPRC: Peter Kochunov (ms.psychiatry@uth.tmc.edu), Department of Psychiatry and Behavioral Science, University of Texas Health Science Center Houston, Houston, TX
(Adhikari et al., 2019; https://doi.org/10.1002/hbm.24723)

Human research participants

Policy information about studies involving human research participants and Sex and Gender in Research.

Reporting on sex and gender

Our findings apply to both male and female sexes. For each referenced dataset (COBRE, FBIRN, and MPRC), sex was based on self-reported demographic assessment. For the present study, we sought to assess differences between aggregated HC and SZ cohorts. Therefore, we used a general linear model (GLM) approach to control for sex as a covariate in all between-subjects analyses.

Population characteristics

Case (SZ) / Control (HC):
193 / 315

Age (years) mean / standard deviation / median / range (minimum - maximum):
38.48 / 12.94 / 39 / (13 - 68)

Sex (female / male):
169 / 339

Race (mixed American (AMR) / European (EUR) / African (AFR) / other):
109 / 304 / 90 / 5

Recruitment

The resting-state fMRI data analyzed in this study was sourced from three case-control psychosis projects: Center for Biomedical Research Excellence (COBRE), Functional Imaging Biomedical Informatics Research Network (FBIRN), and Maryland Psychiatric Research Center (MPRC).

COBRE individuals with schizophrenia (SZ) were recruited from the Raymond G. Murphy Veterans Affairs Medical Center and from psychiatric clinics in metropolitan Albuquerque, New Mexico (Aine et al., 2017). SZ individuals received a diagnosis of schizophrenia performed in consensus by two research psychiatrists via the Structured Clinical Interview for DSM-IV Axis I Disorders (SCID) using the patient version of the SCID-DSM-IV-TR. SZ subjects were evaluated for comorbidities and for retrospective as well as prospective clinical stability. Additional exclusion criteria were as follows: history of neurological disorder, head trauma with loss of conscious exceeding five minutes, mental retardation, or history of active substance dependence or abuse (except nicotine). Healthy control (HC) individuals from the same geographic location were recruited via Institutional Review Board-approved advertisements and completed the SCID-Non-Patient Edition to exclude individuals with Axis I conditions (Aine et al., 2017). Additional exclusion criteria were as follows: current or past psychiatric disorder with the exception of one lifetime major depressive episode, head trauma with loss of conscious exceeding five minutes, recent history of substance abuse or dependence, occurrence of depression or antidepressant use within the past 6 months, history of antidepressant use exceeding one year, and history of psychotic disorder in any first-degree relative. Individuals did not smoke for at least one hour prior to scanning.

FBIRN individuals were recruited across seven different sites within the United States (Damaraju et al., 2014; https://doi.org/10.1016/j.nicl.2014.07.003). SZ individuals were diagnosed with schizophrenia based on the SCID-DSM-IV-TR and

were clinically stable and on antipsychotic medication for at least two months prior to scanning. Exclusion criteria for individuals were as follows: history of major medical illness, MRI contraindications, poor vision with MRI-compatible corrective lenses, IQ under 75, current substance abuse disorder or a history of drug dependence in the last 5 years, extrapyramidal symptoms (for SZ individuals), and current or past history of major neurological or psychiatric illness (SCIS-I/NP) or first-degree relative with Axis I psychotic disorder (for HC).

MPRC individuals with SZ were recruited from outpatient clinics at the Maryland Psychiatric Research Center and mental health clinics in the greater Baltimore area between 2004 and 2016, and HC individuals were recruited via advertisements in the same geographic location (Adhikari et al., 2019). For SZ individuals, a diagnosis of schizophrenia was confirmed via the SCID-DSM-IV. Exclusion criteria for SZ individuals were as follows: major medical or neurological illness, history of head trauma with cognitive sequelae, and diagnosis of intellectual disability. HC exclusion criteria included a past or present diagnosis of DSM-IV Axis I disorder or family history of psychosis in two prior generations.

Case and control participants were compensated for interviews, scan sessions, and assessments conducted during the referenced studies.

Ethics oversight

Subjects provided informed written consent as required and approved by the Institutional Review Boards (IRBs) of the corresponding institutions as follows:

COBRE: participants gave written informed consent as required and approved by the IRB of the University of New Mexico (Aine et al., 2017).

FBIRN: participants gave written informed consent as required and approved by the IRBs of the University of California Irvine, the University of California Los Angeles, the University of California San Francisco, Duke University, University of North Carolina, University of New Mexico, University of Iowa, and University of Minnesota (Damaraju et al., 2014).

MPRC: participants gave written informed consent as required and approved by the IRB of the University of Maryland, Baltimore (Adhikari et al., 2019).

Note that full information on the approval of the study protocol must also be provided in the manuscript.

Field-specific reporting

Please select the one below that is the best fit for your research. If you are not sure, read the appropriate sections before making your selection.

☒ Life sciences ☐ Behavioural & social sciences ☐ Ecological, evolutionary & environmental sciences

For a reference copy of the document with all sections, see nature.com/documents/nr-reporting-summary-flat.pdf

Life sciences study design

All studies must disclose on these points even when the disclosure is negative.

Sample size

This study was conducted on resting-state fMRI data collected from three large multi-site datasets. After quality control criteria were used for subject exclusion, the final subject pool included 315 HC and 193 SZ (n = 508).

Data exclusions

The following subject quality control criteria (Iraji et al., 2023; https://doi.org/10.1002/hbm.26472) were used for the current study: 1) completeness of demographic information, 2) availability of T1 structural MRI, 3) availability of genomic information, 4) maximum head rotation less than 3°, 5) maximum translation less than 3 mm, 6) mean framewise displacement (FD) less than 0.25, 7) quality registration to an echo-planar imaging template, 8) whole-brain (in addition to the top ten and bottom ten slices) spatial overlap between the subject mask and group mask greater than 80%, and 9) removal of duplicate subjects.

Replication

No replication analysis was conducted for the present study.

Randomization

Participants were organized into case and control groups based on SZ diagnosis (see Recruitment). Between-subjects analyses controlled for covariates including age, sex, site, and motion (mean framewise displacement) using a GLM approach.

The independent component analysis (ICA) approach to network estimation included a bootstrapped randomization protocol for each FC metric-specific analysis to ensure component reliability across runs. For the unique ENL ICN validation protocol, ICA was conducted on subsets of 80% of subjects drawn from the total subject pool with five bootstrapped runs per analysis. Statistical randomization (permutation) tests were conducted with 5000 random permutations to assess differences in goodness-of-fit between HC and SZ, differences in estimation reliability between ENL and LIN components, and the robustness of voxel-wise t-tests.

Blinding

No blinding procedure was conducted for the present analysis of the referenced datasets.

Reporting for specific materials, systems and methods

We require information from authors about some types of materials, experimental systems and methods used in many studies. Here, indicate whether each material, system or method listed is relevant to your study. If you are not sure if a list item applies to your research, read the appropriate section before selecting a response.

Materials & experimental systems

n/a	Involved in the study
☒	☐ Antibodies
☒	☐ Eukaryotic cell lines
☒	☐ Palaeontology and archaeology
☒	☐ Animals and other organisms
☒	☐ Clinical data
☒	☐ Dual use research of concern

Methods

n/a	Involved in the study
☒	☐ ChIP-seq
☒	☐ Flow cytometry
☐	☒ MRI-based neuroimaging

Magnetic resonance imaging

Experimental design

Design type: Resting-state fMRI

Design specifications: No task-based fMRI data were included in the present study

Behavioral performance measures: No task-based fMRI data were included in the present study

Acquisition

Imaging type(s): Functional

Field strength: 3T

Sequence & imaging parameters: COBRE data were collected at a single site on a Siemens TIM Trio scanner via an echo-planar imaging sequence (TR = 2000 ms; TE = 29 ms) (Iraji et al., 2022; https://doi.org/10.1162/netn_a_00196). Voxel spacing was 3.75 x 3.75 x 4.5 mm, the slice gap was 1.05 mm, and the field of view (FOV) was 240 x 240 mm. FBIRN data were collected from seven sites (Turner et al., 2013; https://doi.org/10.3389/fnins.2013.00137), with six sites utilizing Siemens TIM Trio scanners and one utilizing a General Electric Discovery MR750 (Iraji et al., 2022). All seven sites used an echo-planar imaging sequence (TR = 2000 ms; TE = 30 ms). Original voxel spacing was 3.4375 x 3.4375 x 4 mm, the slice gap was 1 mm, and the FOV was 220 x 220 mm. MPRC data were collected from three sites via echo-planar imaging sequences (Iraji et al., 2022). One site used a Siemens Allegra scanner (TR = 2000 ms; TE = 27 ms; voxel spacing = 3.44 x 3.44 x 4 mm; FOV = 220 x 220 mm), another used a Siemens TIM Trio scanner (TR = 2210 ms; TE = 30 ms; voxel spacing = 3.44 x 3.44 x 4 mm; FOV = 220 x 220 mm), and the third site used a Siemens TIM Trio scanner (TR = 2000 ms; TE = 30 ms; voxel spacing = 1.72 x 1.72 x 4 mm; FOV = 220 x 220 mm).

Area of acquisition: COBRE field of view (FOV): 240 x 240 mm; FBIRN FOV: 220 x 220 mm (all sites); MPRC FOV: 220 x 220 mm (all sites)

Diffusion MRI ☐ Used ☒ Not used

Preprocessing

Preprocessing software: MATLAB, FSL v6.0, SPM 12

Normalization: Nonlinear

Normalization template: EPI template

Noise and artifact removal: Preprocessing was performed primarily within the MATLAB software environment using Statistical Parametric Mapping (SPM12; http://www.fil.ion.ucl.ac.uk/spm/) and the FMRIB Software Library (FSL v6.0; https://fsl.fmrib.ox.ac.uk/fsl/fslwiki). Preprocessing steps included 1) rigid body motion and slice timing correction, 2) nonlinear warping to Montreal Neurological Institute (MNI) 152 coordinate space, 3) spatial resampling to 3 mm isotropic voxel spacing, 4) spatial smoothing with a 6 mm full width at half maximum (FWHM) Gaussian kernel, 5) head motion regression, detrending, despiking, low pass filtering, 6) temporal resampling to TR = 2000 ms, and finally 7) voxel time series Z-scoring to normalize variance.

After the implementation of group-level ICA, components were screened and considered to be artifactual if they exhibited an ICASSO IQ value less than .80, 2) exhibited low visual overlap with gray matter, 3) exhibited peak weight outside of white matter, and 4) exhibited high visual similarity to motion, ventricular, and other known artifacts (Iraji et al., 2023). The subject-level components corresponding to the non-artifactual group-level components were used for further analysis.

Volume censoring: The following subject quality control criteria (Iraji et al., 2023) were used for the current study: 1) completeness of demographic information, 2) availability of T1 structural MRI, 3) availability of genomic information, 4) maximum head rotation less than 3°, 5) maximum translation less than 3 mm, 6) mean framewise displacement (FD) less than 0.25, 7) quality

registration to an echo-planar imaging template, 8) whole-brain (in addition to the top ten and bottom ten slices) spatial overlap between the subject mask and group mask greater than 80%, and 9) removal of duplicate subjects.

Statistical modeling & inference

Model type and settings

Independent component analysis (ICA), univariate parametric and non-parametric analysis, multivariate modeling to balance HC and SZ cohorts for covariates.

Effect(s) tested

No task-based fMRI data were analyzed. The present study tested for the effect of SZ diagnosis on regression of nonlinear whole-brain functional connectivity (NL-wFC) on linear whole-brain functional connectivity (LIN-wFC) goodness-of-fit, effect of explicitly nonlinear (ENL) vs. linear (LIN) connectivity metrics on independent component analysis component estimation reliability (ICASSO IQ), effect of ENL vs. LIN on intrinsic connectivity network (ICN) voxel weight, effect of SZ diagnosis on ENL ICN voxel weight, and effect of SZ diagnosis on LIN ICN voxel weight. We considered age, sex, site, and motion (mean framewise displacement) to be confounding factors and used a GLM approach to account for their effects in all between-subjects statistical analyses.

Specify type of analysis: ☐ Whole brain ☐ ROI-based ☒ Both

Anatomical location(s)

We used the Group ICA of fMRI Toolbox (GIFT v4.0; http://trendscenter.org/software/gift) to implement connectivity domain ICA (Iraji et al., 2016; https://doi.org/10.1016/j.neuroimage.2016.04.006) and obtain separate sets of group-level brain networks from linear whole-brain functional connectivity (LIN-wFC) and explicitly nonlinear whole-brain functional connectivity (ENL-wFC) data. The implementation of group-level spatial independent component analysis (gr-sICA) was preceded by an initial subject-level multi-power iteration (Rachakonda et al., 2016; https://doi.org/10.3389/fnins.2016.00017) principal component analysis step to reduce dimensionality and denoise the data (Erhardt et al., 2011; https://doi.org/10.1002/hbm.21170). The 30 principal components that explained the maximum variance of each subject's respective LIN-wFC and ENL-wFC were retained for further analysis. Subject-level principal components from each estimator were concatenated across the component dimension, and a group-level principal component analysis step was applied to further reduce the dimensionality of the data and decrease the computational demands of gr-sICA (Calhoun et al., 2009; https://doi.org/10.1016/j.neuroimage.2008.10.057). The 20 group-level principal components that explained the maximum variance of each estimator-specific data set were used as the input for gr-sICA. We selected a gr-sICA model order of 20 to obtain large-scale functional networks (Iraji et al., 2016; Ray et al., 2013; https://doi.org/10.3389/fnins.2013.00237). To ensure the reliability of our results, ICA was implemented via the Infomax optimization algorithm (Bell & Sejnowski, 1995; https://doi.org/10.1162/neco.1995.7.6.1129) 100 times with both random initialization and bootstrapping, and the most stable run was selected for further analysis. We evaluated the reliability and quality of ENL and LIN components using the ICASSO quality index (IQ), which quantifies component stability across runs (Himberg et al., 2004; https://doi.org/10.1016/j.neuroimage.2004.03.027). To assess the difference in stability between ENL and LIN components, we conducted a two-sided permutation test with 5000 random permutations on the IQ data. Assessing component reliability was a necessary step, as previous work demonstrates that certain components may be inconsistently extracted from the data of interest (Himberg et al., 2004). In the context of fMRI network estimation, ICASSO IQ is often used to differentiate reliable components from components that are unstable and unfit for further analysis (Iraji et al., 2019; https://doi.org/10.1002/hbm.24580). A component was identified as an ICN if and only if 1) it exhibited an ICASSO IQ value exceeding .80, 2) it exhibited high visual overlap with gray matter, 3) it exhibited peak weight within gray matter, and 4) it exhibited low visual similarity to motion, ventricular, and other known artifacts. To find spatially corresponding networks, the spatial correlation value was computed between every pair of extracted LIN and ENL components, and components were matched in a greedy fashion. ICNs matched with a spatial correlation value exceeding .80 were classified as common (Iraji et al., 2023) and were labeled based on their neuroanatomical distributions and the identification of ICNs from previous studies (Iraji et al., 2016). Networks exhibiting maximum spatial correlation less than .40 were classified as unique. We used the Group ICA of fMRI Toolbox (GIFT v4.0) to implement group information-guided ICA (GIG-ICA) (Du & Fan, 2013; https://doi.org/10.1016/j.neuroimage.2012.11.008) and reconstruct subject-specific networks from subject-level principal components using the group-level spatial references.

To assess differences in spatial variation between matched networks, we conducted voxel-wise two-sided paired samples t-tests on their Z-scored subject-level estimates. For a given matched network pair, statistical comparisons were masked for voxels exceeding Z = 1.96 (p = .05) in either group-level map (LIN or ENL), and the False Discovery Rate (FDR) method was used to correct for multiple comparisons (q < .05) (Benjamini & Hochberg, 1995; http://www.jstor.org/stable/2346101). The robustness of the voxel-wise t-test procedure was assessed via comparison to the results of voxel-wise two-sided permutation tests with 5000 random permutations for the posterior default mode (pDM) network. The automated anatomical labeling atlas 3 (AAL3) (Rolls et al., 2020; https://doi.org/10.1016/j.neuroimage.2019.116189) was used to localize clusters of significant voxels to anatomically defined brain regions.

To assess differences between HC and SZ, we conducted voxel-wise two-sided independent samples t-tests between the estimates of common and unique networks derived from each cohort. We first used a GLM to remove the effect of confounding factors including age, sex, site, and motion (mean framewise displacement) on Z-scored subject-level network estimates. Voxel-wise independent samples two-sided t-tests were then conducted on the residual spatial maps derived from the HC and SZ groups. Statistical comparisons between common networks were masked for voxels exceeding Z = 1.96 (p = .05) in either of the group-level maps (LIN or ENL), while unique network comparisons were masked for voxels exceeding the same threshold in the unique group-level map. The FDR method (Benjamini & Hochberg, 1995) was used to correct for multiple comparisons (q < .05). The robustness of the voxel-wise t-test procedure was assessed via comparison to the results of voxel-wise two-sided permutation tests with 5000 random permutations for the posterior default mode (pDM) network. The AAL3 atlas (Rolls et al., 2020) was used to localize clusters of significant voxels to anatomically defined brain regions. A two-sided McNemar's test was used to assess the overall ENL vs. LIN difference in statistical sensitivity (across all voxels belonging to commonly classified networks), and differences in statistical sensitivity for matched network pairs were investigated separately using either two-sided McNemar's tests or exact binomial tests (for n < 25).

Statistic type for inference
(See Eklund et al. 2016)

Voxel-wise

Correction

FDR (Benjamini & Hochberg, 1995; http://www.jstor.org/stable/2346101)

Models & analysis

n/a	Involved in the study
☐	☒ Functional and/or effective connectivity
☒	☐ Graph analysis
☐	☒ Multivariate modeling or predictive analysis

Functional and/or effective connectivity

Pearson correlation (to construct LIN-wFC), distance correlation (to construct NL-wFC), explicitly nonlinear whole-brain functional connectivity (ENL-wFC)

Multivariate modeling and predictive analysis

We used the Group ICA of fMRI Toolbox (GIFT v4.0; http://trendscenter.org/software/gift) to implement connectivity domain ICA (Iraji et al., 2016) and obtain separate sets of group-level brain networks from linear whole-brain functional connectivity (LIN-wFC) and explicitly nonlinear whole-brain functional connectivity (ENL-wFC) data. The implementation of group-level spatial independent component analysis (gr-sICA) was preceded by an initial subject-level multi-power iteration (Rachakonda et al., 2016) principal component analysis step to reduce dimensionality and denoise the data (Erhardt et al., 2011). The 30 principal components that explained the maximum variance of each subject's respective LIN-wFC and ENL-wFC were retained for further analysis. Subject-level principal components from each estimator were concatenated across the component dimension, and a group-level principal component analysis step was applied to further reduce the dimensionality of the data and decrease the computational demands of gr-sICA (Calhoun et al., 2009). The 20 group-level principal components that explained the maximum variance of each estimator-specific data set were used as the input for gr-sICA. We selected a gr-sICA model order of 20 to obtain large-scale functional networks (Iraji et al., 2016; Ray et al., 2013). To ensure the reliability of our results, ICA was implemented via the Infomax optimization algorithm (Bell & Sejnowski, 1995) 100 times with both random initialization and bootstrapping, and the most stable run was selected for further analysis. We evaluated the reliability and quality of ENL and LIN components using the ICASSO quality index (IQ), which quantifies component stability across runs (Himberg et al., 2004). To assess the difference in stability between ENL and LIN components, we conducted a two-sided permutation test with 5000 random permutations on the IQ data. Assessing component reliability was a necessary step, as previous work demonstrates that certain components may be inconsistently extracted from the data of interest (Himberg et al., 2004). In the context of fMRI network estimation, ICASSO IQ is often used to differentiate reliable components from components that are unstable and unfit for further analysis (Iraji et al., 2019). A component was identified as an ICN if and only if 1) it exhibited an ICASSO IQ value exceeding .80, 2) it exhibited high visual overlap with gray matter, 3) it exhibited peak weight within gray matter, and 4) it exhibited low visual similarity to motion, ventricular, and other known artifacts. To find spatially corresponding networks, the spatial correlation value was computed between every pair of extracted LIN and ENL components, and components were matched in a greedy fashion. ICNs matched with a spatial correlation value exceeding .80 were classified as common (Iraji et al., 2023) and were labeled based on their neuroanatomical distributions and the identification of ICNs from previous studies (Iraji et al., 2016). Networks exhibiting maximum spatial correlation less than .40 were classified as unique. We used the Group ICA of fMRI Toolbox (GIFT v4.0) to implement group information-guided ICA (GIG-ICA) (Du & Fan, 2013) and reconstruct subject-specific networks from subject-level principal components using the group-level spatial references.

For the supplementary analysis of balanced HC and SZ cohorts, we used multivariate genetic matching with replacement (Sekhon et al., 2011; https://doi.org/10.18637/jss.v042.i07) to balance cohorts for confounding factors including age, sex, site, and motion (mean framewise displacement).

March 2021
